# Postnatal development in a marsupial model, the fat-tailed dunnart (*Sminthopsis crassicaudata*; Dasyuromorphia: Dasyuridae)

**DOI:** 10.1038/s42003-021-02506-2

**Published:** 2021-09-02

**Authors:** Laura E. Cook, Axel H. Newton, Christy A. Hipsley, Andrew J. Pask

**Affiliations:** 1 School of Biosciences, University of Melbourne, Parkville, VIC Australia; 2 Department of Anatomy and Developmental Biology, Monash University, Clayton, VIC Australia; 3 Department of Biology, University of Copenhagen, Copenhagen, Denmark; 4 Department of Sciences, Museums Victoria, Carlton, VIC Australia

**Keywords:** Embryogenesis, Embryology, Evolutionary developmental biology

## Abstract

Marsupials exhibit unique biological features that provide fascinating insights into many aspects of mammalian development. These include their distinctive mode of reproduction, altricial stage at birth, and the associated heterochrony that is required for their crawl to the pouch and teat attachment. Marsupials are also an invaluable resource for mammalian comparative biology, forming a distinct lineage from the extant placental and egg-laying monotreme mammals. Despite their unique biology, marsupial resources are lagging behind those available for placentals. The fat-tailed dunnart (*Sminthopsis crassicaudata*) is a laboratory based marsupial model, with simple and robust husbandry requirements and a short reproductive cycle making it amenable to experimental manipulations. Here we present a detailed staging series for the fat-tailed dunnart, focusing on their accelerated development of the forelimbs and jaws. This study provides the first skeletal developmental series on *S. crassicaudata* and provides a fundamental resource for future studies exploring mammalian diversification, development and evolution.

## Abstract (web summary)

Cook, Pask and colleagues describe the ossification of the skull and forelimbs in the developing postnatal fat-tailed dunnart. As one of the most altricial marsupials at birth with a short reproductive cycle and simple lab husbandry, this species is a promising model organism for jaw and forelimb development in mammals.

## Introduction

**Fig. 1:**
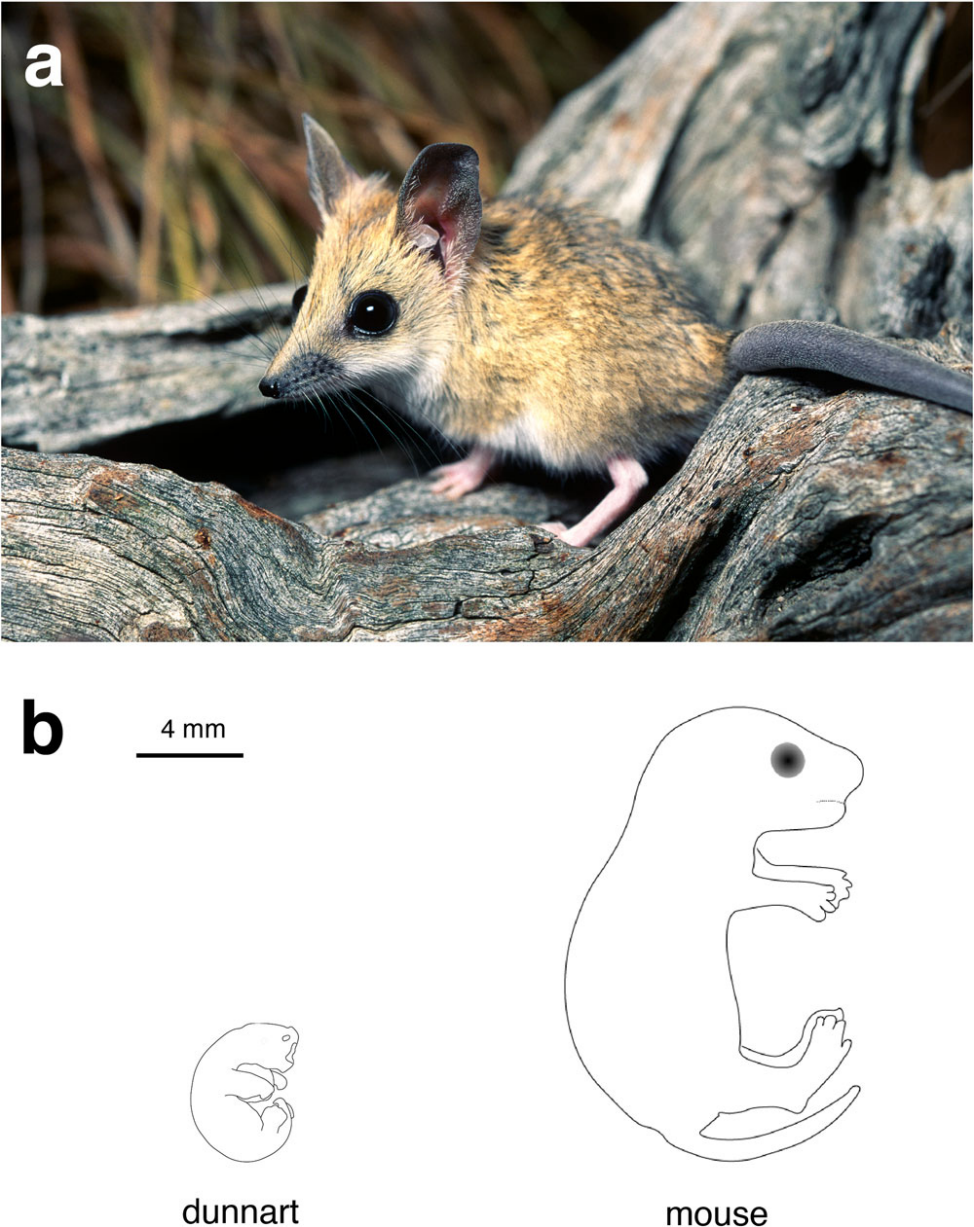
At birth the fat-tailed dunnart represents one of the most altricial mammals in existence. **a** Adult fat-tailed dunnart (*S. crassicaudata*). Image: Alan Henderson—Minibeast Wildlife. **b** Schematic comparing extent of development of the fat-tailed dunnart and mouse (*M. musculus*) neonates on the day of birth.

Marsupials (Metatheria) represent a distinct viviparous lineage within extant Mammalia that have been evolving independently from their sister group placental mammals (Eutheria) for over 160 million years^1,2^. This evolutionary distance has made them exceptionally powerful in comparative biology for understanding mammalian evolution on both a genetic and developmental level. In addition to their use for comparative studies, marsupials also exhibit unique biological features, chief among them is their unusual mode of reproduction. Eutherian mammals (hereafter referred to as placentals) typically give birth to well-developed young after a prolonged period of gestation with a large maternal contribution to the development of the offspring in utero. In contrast, marsupials give birth to highly altricial young (Fig. 1b) after a short gestation with only a minimal maternal investment during pregnancy via a short-lived, simple placenta^3^. Instead, marsupials have a large maternal contribution post-birth where the young are dependent on maternal milk through an extended lactation period^3,4^. Newborn marsupials crawl to the mother’s teat, typically located within her pouch, where they undergo the majority of their development, *ex utero*^4–6^. To facilitate the crawl to the teat, marsupials have well-developed forelimbs and shoulder girdles, but comparatively delayed development of the hindlimbs^7–12^. Similarly, craniofacial structures such as the nasal cavity, tongue, oral bones and musculature are accelerated relative to the development of the posterior end of the body^13–15^. Chondrification and ossification of the facial skeleton, forelimbs and shoulder girdle are significantly accelerated in marsupials compared to placentals^13,14,16^. This heterochrony is suggested to underpin developmental constraints in the marsupial clade that have restricted their overall morphological diversity within the limb and facial skeleton compared with placental mammals^17,18^, though this remains controversial^19,20^. Defining the mechanisms that control these unique developmental events can therefore provide insights into the processes underlying limb and craniofacial patterning across mammals.

Traditionally, the marsupial models, *Macropus eugenii* (tammar wallaby) and *Monodelphis domestica* (opossum), have been studied in regard to diverse biological phenomena, including sex determination^21–28^, reproduction^29–34^, genomic imprinting^35–39^ and other aspects of development^40–46^. Given their ease of captive breeding and experimental manipulation, *M. domestica* has been used in research across North America, for example to model craniofacial phenotypes observed in children treated with thalidomide^47^, and to reconstruct the evolution of the mammalian middle ear^48^. However, the 80-million-year divergence between Australian and North American marsupials^49^ means that an Australian laboratory-based marsupial model with similarly easy husbandry, year-round breeding and experimental manipulation is still needed for a more complete understanding of mammalian biology. The fat-tailed dunnart (*Sminthopsis crassicaudata*; hereafter referred to as the dunnart) is an established marsupial model that has been used successfully for studies of brain development, fertilisation, reproduction, respiration, nutrition, thermoregulation, vision and immunology^50–56^. In this context, expansion of the developmental and genetics resources available for the dunnart would further its value as a model for investigating mammalian biology and evolution.

**Fig. 2:**
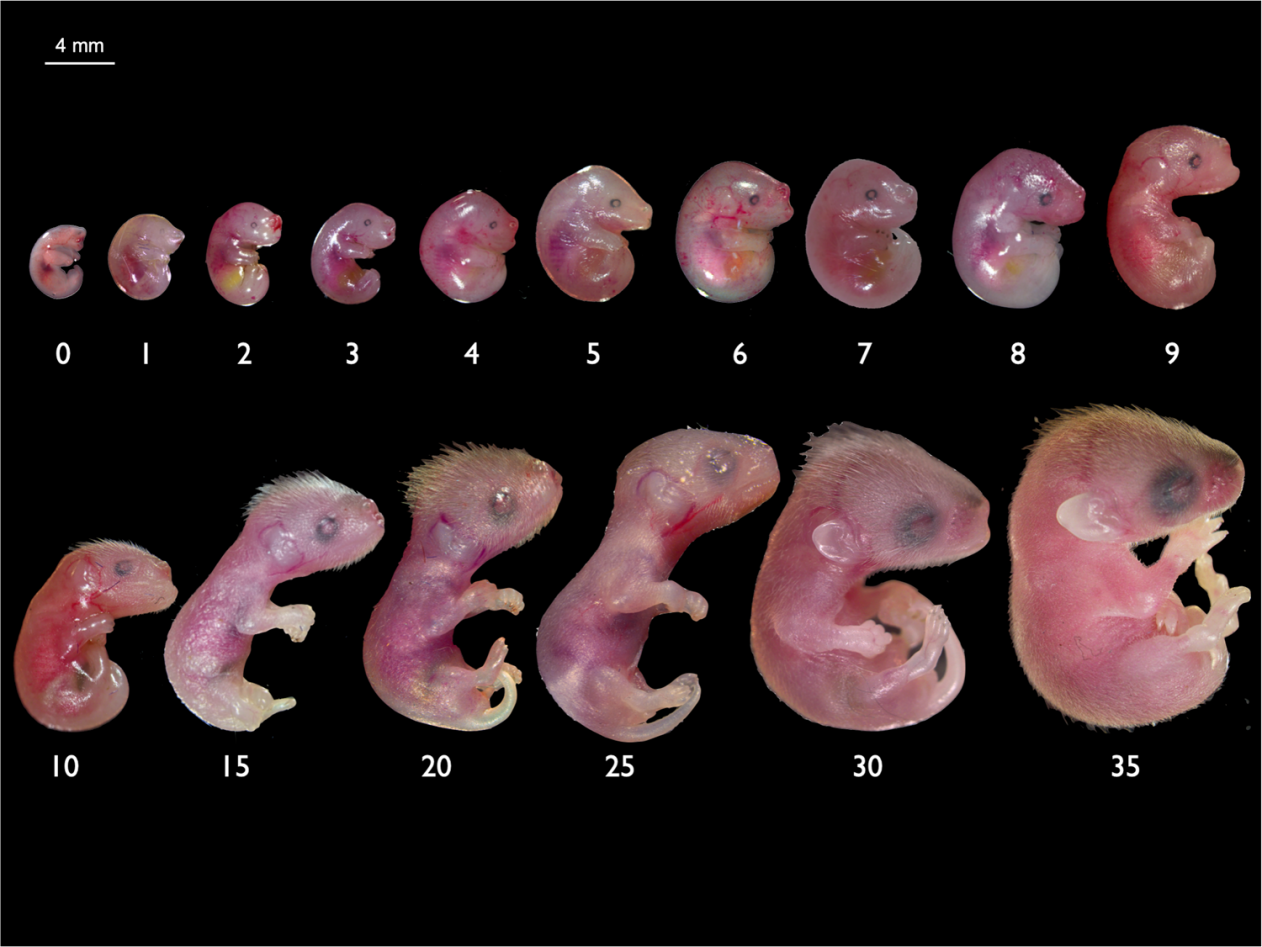
Gross morphology of the fat-tailed dunnart throughout postnatal development in the pouch. Numbers below pouch young refer to the day postpartum.

Fat-tailed dunnarts are small, carnivorous Australian marsupials from the family Dasyuridae that contains about 70 other living species (Fig. 1a). Dunnarts have a short gestation of 13.5 days and represent one of the most altricial mammals known^8^ (Figs. 1b and 2). They typically give birth to supernumerary young^57^ and can suckle up to 10 young (equal to the number of teats). Juvenile dunnarts are weaned after ~65–70 days postpartum (D)^50,58^, with males reaching sexual maturity at approximately D200 and females entering their first oestrus at approximately D85^58^. The dunnart can breed all year round, which allows for efficient expansion and maintenance of colony numbers and derivation of staged foetuses and pouch young for experimental studies. As such, the dunnart provides an excellent opportunity to study and manipulate early mammalian development, which occurs almost entirely *ex utero*.

Detailed staging series are imperative resources for facilitating the study of any model species, but are especially interesting in marsupials owing to their unique developmental biology. There are currently no published data on the development of the skeleton of the fat-tailed dunnart, and studies on other related marsupials are often from whole-mount bone and cartilage staining on limited stages^59–62^, or X-ray computed tomography (CT) scanning of museum specimens where absolute age is typically unknown^63–66^. Previous histological studies of ossification patterns in marsupials have suggested that in highly altricial species, ossification occurs after birth^60–62^. If ossification of the dunnart skeleton also occurs post-birth, observations and manipulations of early skeletal patterning and development can be made postnatally in the pouch, further supporting its use as a model organism. However, a detailed description and staging of these events in the dunnart has yet to be established.

Here, we establish a complete postnatal ontogenetic series of the dunnart, emphasizing critical stages underlying the onset and early ossification of the cranial and postcranial skeleton. We provide quantitative developmental comparisons of craniofacial and limb heterochrony and development compared to the established placental mouse model, *Mus musculus*. This developmental series lays the foundation for further studies into the molecular and genetic control of the many unique aspects of marsupial development including mechanisms underlying extreme skeletal heterochrony. Our findings complement the ongoing development of additional *S. crassicaudata* resources as a marsupial laboratory model, including gold standard genetic resources, such as a chromosome level genome, inbred laboratory strains, and induced pluripotent cell lines.

## Results

### Gross morphology

**Fig. 3:**
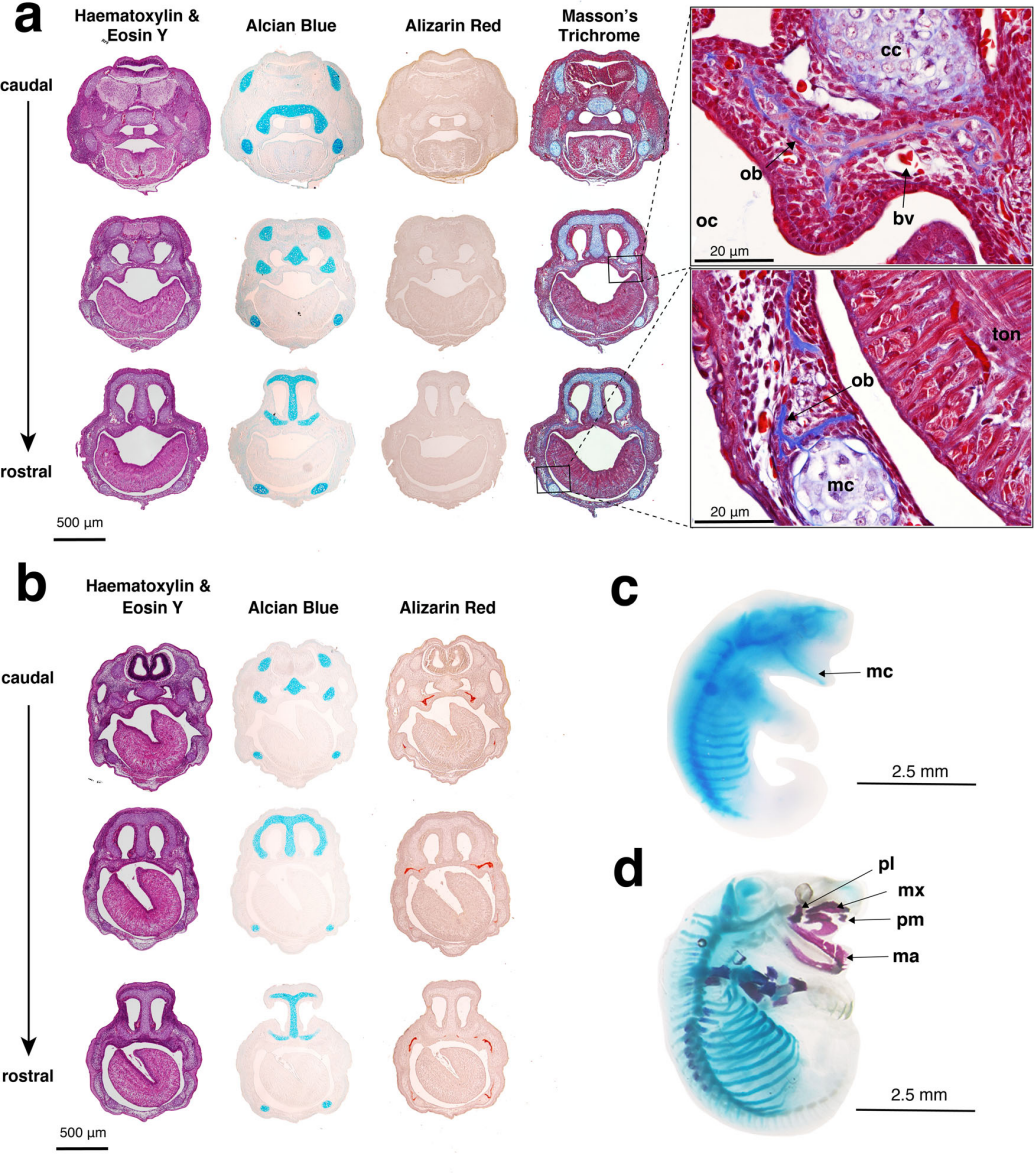
Histological analyses were used to determine ossification onset in D0 and D1 fat-tailed dunnart pouch young. **a** D0 head sections stained with haematoxylin and eosin Y (purple = nuclei, pink = cytoplasm), alcian blue (cartilage), alizarin red (mineralised bone) and Masson’s trichrome (blue = collagen, red = muscles, cytoplasm, and keratin). Representative images of D0 at higher magnification (×60) show ossification had begun with collagen present in the maxillary and mandible prominences. **b** D1 head sections stained with haematoxylin and eosin Y (purple = nuclei, pink = cytoplasm), alcian blue (cartilage), and alizarin red (mineralised bone). Wholemount, **c** D0 and **d** D1 fat-tailed dunnart specimens cleared and stained with alcian blue (cartilage) and alizarin red (bone). bv = blood vessel, cc = cartilago cupularis, ma = mandible, mc = Merkel’s cartilage, mx = maxillary, ob = osteoblast, oc = oral cavity, pl = palatine process, ton = tongue.

Newborn dunnarts (D0) had an average head length of 2 mm and average weight of 12 mg (Supplementary Table 2). They were bright pink at birth and had highly vascularised, translucent skin with the developing lungs, heart and bladder clearly visible through the skin. Newborns lacked a definitive neck and had a pronounced cervical swelling under the head between the forelimbs, which disappeared by D4. At birth there were no ear primordia, and very faint retina pigmentation was visible indicating the location of the future eyes (Fig. 2, Supplementary Fig. 1a). At birth the dorsal side of the neurocranium was flat and did not begin to round until 24 h after birth. The mandibular and maxillary swellings were visible along with the hyoid/secondary arch. The medial nasal swelling was the dominant feature of the face and contributed to a large prominent nostril (Supplementary Fig. 2). This stage in the development of the facial prominences is comparable to the mouse on embryonic day (E) 11.5–12^67^ (Supplementary Fig. 2). On the day of birth, the oral region was completely fused except for a small round opening for attachment to the teat. By D15 the mouth began to delaminate and was fully separated by D30, coinciding with tooth eruption (Supplementary Fig. 3a). Dunnart limb heterochrony was distinct, where on D0 forelimbs were well developed (including cartilaginous skeletal elements and musculature) with interdigital separation and claws present, while the hindlimbs were rudimentary and paddle-like with faint digital grooves. The proximal region of the hindlimb remained fused with the base of the tail until approximately D9. There were no cartilaginous condensations in the dunnart hindlimb at D0 or D1 (Fig. 3c, d), comparable to the E11.5 mouse hindlimb^68^. The newborn fat-tailed dunnart forelimbs appear similar to those of the E14–E15 mouse with regards to digit development^68^. However, in the mouse forelimb this coincides with the presence of forelimb ossification centres^69^, which are not present in dunnart newborns. Unlike diprotodontian marsupials (marsupials with forward facing pouches, order Diprotodontia) which display strong climbing motion of the forelimbs at birth^4,6,70–72^, on D0 the dunnart appeared to have a weaker climbing motion after being removed from the pouch (Supplementary Movie 1).

At birth marsupials display sexual dimorphisms which precede gonad development and are under the control of the X chromosome. The appearance of the pouch and scrotum are determined by the number of X chromosomes present, with one X chromosome determining the development of a scrotum and two X chromosomes leading to the development of a pouch^73^. Female dunnart pouch young display visible mammary primordia from D2 while the scrotal bulges in males are not easily distinguished until D4. Figure 2 shows the gross morphology from D0 to D30. A table of features for staging young is presented in Table 1.

**Table 1:** Features of postnatal development in *S. crassicaudata*.

Age (days post-partum)	Features
0	Bright red, highly vascularised Translucent, hairless skin Developing heart, lungs and bladder visible through the skin No definitive neck Neurocranium flat and the head looks like a tube Cervical swelling present under the mandibular process Very faint retina pigmentation is visible at the location of the future eye Large medial nasal swelling Forelimbs well developed with digit separation and claws Hindlimbs are paddle-like with very faint digital grooves Tail and hindlimbs fused Oral region completely fused except for a small round opening for attachment to the teat
1	Neurocranium becomes rounded
2	Visible pouch primordia in females Forelimb claws pigmented
3	Prominent underbite (mandibular prognathism)
4	Cervical swelling disappears Scrotal bulge visible in males
5	Hindlimb digits with webbing
8	Hair follicles present on top of head
9	Hindlimb no longer fused to tail A thickening in the place of the future ear is distinguishable
10	Hair follicles present on lower jaw
15	Fused mouth begins to delaminate on both sides of the circular opening Hindlimb digits are separated and claws present
20	Hair follicles beginning to grow on body Hindlimb claws pigmented
30	Mouth fully separated Tooth eruption External ear is unfolded
40	Body fully covered in grey/brown fur
60	Testes enclosed in scrotum Eyes beginning to open

Iodine-stained pouch young allow the visualisation of soft tissues using microCT scanning. For the early stages (<D10) the pouch young tissue is very lean, so the internal resolution at these stages is less clear than older specimens. Nevertheless, details of the developing organs can be observed. The major phase of organogenesis in the dunnart occurs almost entirely in the postnatal period, captured here in high resolution (Supplementary Movies 2, 3, 4, 5, 6 and 7). These movies span the major differentiation events for all major organs. The lungs of the neonates were characteristic of primitive airways with few tubular-like structures and limited branching (Supplementary Fig. 1b). Air bubbles under the skin were visible (Supplementary Fig. 1a), consistent with gas exchange occurring via the skin at this early stage. The lung increased in complexity between D30 and D35, with the saccules becoming smaller and increasing the surface area for gas exchange (Supplementary Fig. 3, Supplementary Movies 3 and 4). A large mesonephros was present in the peritoneal cavity at birth (Supplementary Fig. 1b, Supplementary Movie 2). The mesonephros is the functional kidney in most neonatal marsupials^74,75^. By D3 the metanephros (definitive kidney) was visible and by D10, displayed a distinguished renal cortex and medulla. By D20 the mesonephros appeared to have regressed.

Marsupials are one of the few mammals to undergo testicular descent and inguinal closure^31,76^. In our developmental series we were able to capture the process of testicular descent through the inguinal canal and into the scrotum. On D40 the testes had migrated to the base of the abdomen and were ready to begin the inguinoscrotal phase of testicular descent (Supplementary Fig. 4a, Supplementary Movie 5). At D50 the testes were visible in the body wall (Supplementary Fig. 4b, Supplementary Movie 6), transitioning the inguinal canal and by D60 they were situated in the scrotum (Supplementary Fig. 4c, Supplementary Movie 7).

### Onset of ossification in the fat-tailed dunnart skeleton

**Fig. 4:**
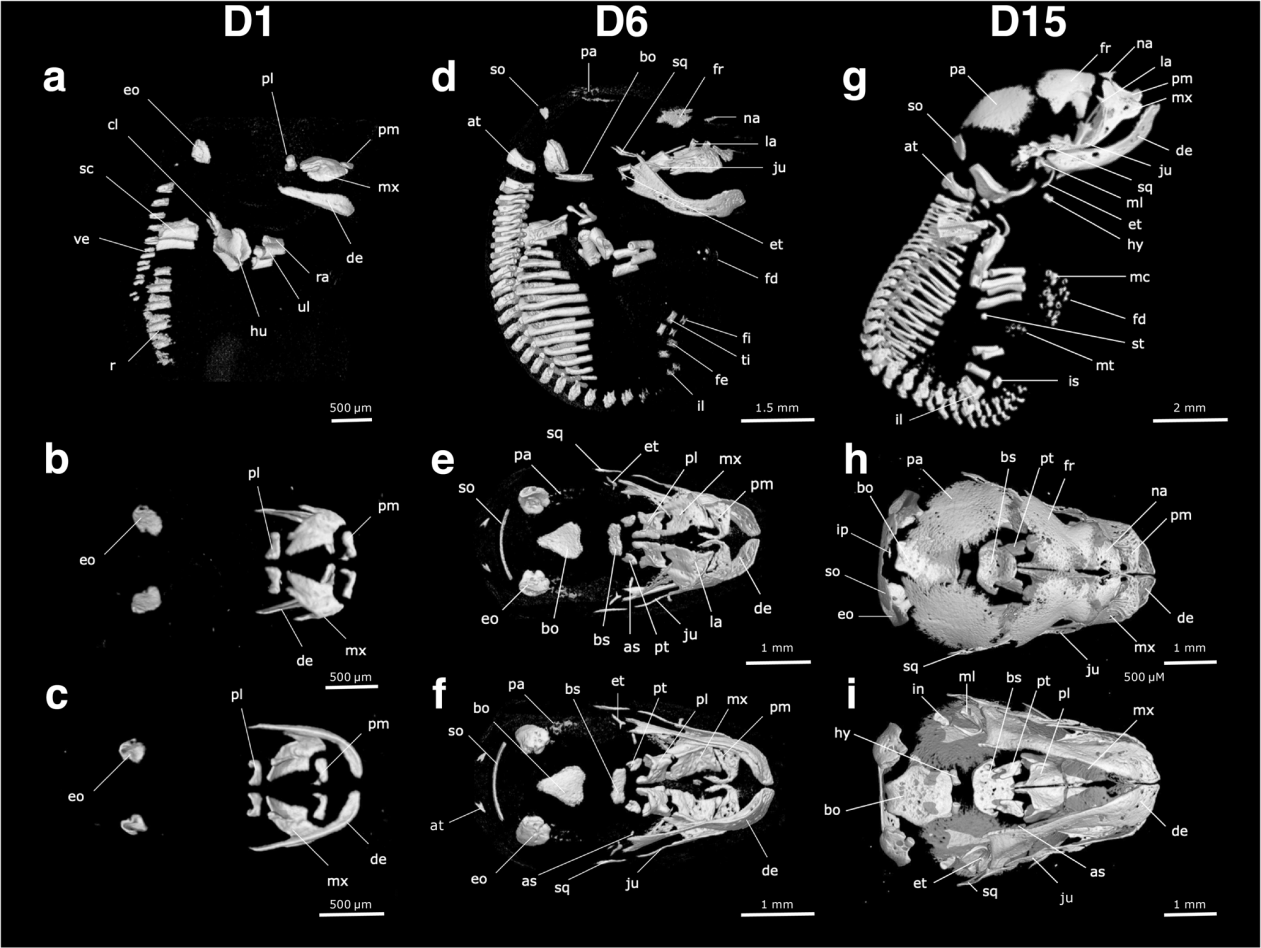
The skeleton of the fat-tailed dunnart (D1, D6 and D15), as revealed in microCT scans of pouch young. The right lateral side of the skeleton is shown in (**a**), (**d**) and (**g**), the dorsal view of the skull in (**b**), (**e**) and (**h**), and the ventral view of the skull in (**c**), (**f**) and (**i**). Pouch young one day after birth (D1) are shown in (**a**), (**b**) and (**c**). Pouch young six days (D6) after birth are shown in (**d**), (**e**) and (**f**). Pouch young 15 days after birth (D15) are shown in (**g**), (**h**) and (**i**). as = alisphenoid, at = atlas, bo = basioccipital, bs = basisphenoid, cl = clavicle, de = dentary, eo = exooccipital, et = ectotympanic, fd = forelimb digits, fe = femur, fi = fibula, fr = frontal, hu = humerus, hy = hyoid, in = incus, il = ilium, ip = interparietal, is = ischium, ju = jugal, la = acrimal, mc = metacarpals, ml = malleus, mt = metatarsals, mx = maxilla, na = nasal, pa = parietal, pl = palatine process, pm = premaxilla, pt = pterygoid, ra = radius, ri = ribs, sc = scapula, so = supraoccipital, sq = squamosal, st = sternum, ti = tibia, ul = ulna, ve = vertebrae.

At birth, pouch young lack an ossified skeleton, showing a cartilaginous postcranial skeleton and chondrocranium (Fig. 3a). Positive alizarin red staining, which stains mineralised bone, was observed in the maxillary and dentary in D1 frontal sections but not at D0 (Fig. 3a, c). However, when stained with Masson’s trichrome stain, collagen deposits (blue) typical of osteoid matrix and bone were present in the maxillary and dentary tissue (Fig. 3a) suggesting that ossification had started in the newborn pouch young. Within 24 h of birth, ossified bones of the facial and postcranial skeleton were present as observed with wholemount alizarin red staining (Fig. 3d) and microCT scanning (Fig. 4a).

### Cranial ossification

On D1, ossification centres were evident in the premaxilla, maxilla, palatine process and exoccipital bones (Fig. 4a–c). The pterygoid and basioccipital bones were observed on D3. The first ossification centre of the zygomatic arch began in the jugal on D4, along with the basisphenoid and frontal bones of the skull. By D5, ossification centres in the supraoccipital, squamosal, nasal, lacrimal, ectotympanic, goniale and parietal were present. Ossification of the alisphenoid was evident on D6 and the malleus on D7 (Fig.  4d–f). Ossification centres of the incus and interparietal were present on D15 (Fig. 4g–i). The petrosal bone of the inner ear was the last cranial bone to begin ossification and was clearly observable on D20.

**Fig. 5:**
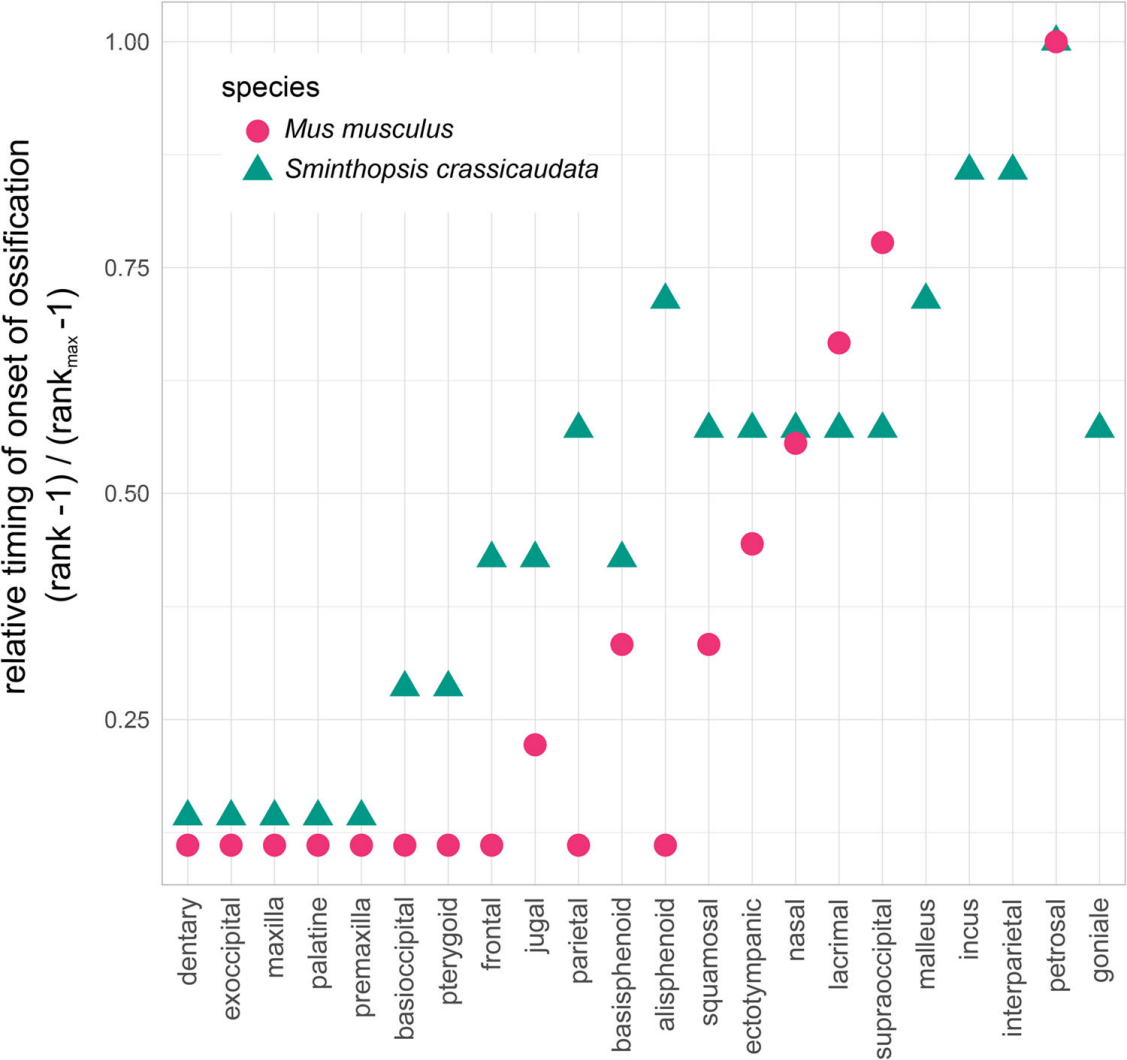
Relative timing of onset of ossification for the bones of the skull in the fat-tailed dunnart (*S. crassicaudata*) and mouse (*M. musculus*). Specimens were ranked in order of bone onset or bone contact timing and relative ranks were normalised for comparison between species. *M. musculus* is shown with pink circles and *S. crassicaudata* is shown with green triangles, highlighting the early onset of ossification of the oral region in both species.

At birth, the lower jaw consists entirely of a thin rod of cartilage, known as Meckel’s cartilage (Fig. 3a, c). By D1 ossification of the dentary bone (mandible) surrounding the Meckel’s cartilage had begun. The coronoid, condylar and angular processes that form as part of the dentary were visible on D4. The oral bones were the first to ossify in both the dunnart and the mouse^77–79^ (Fig. 5). However, the onset of cranial ossification was more uniform in the mouse with bones of the cranium ossifying alongside the oral bones (Fig. 5). In contrast, ossification in the bones of the fat-tailed dunnart cranium occur long after ossification of the oral bones was initiated (Fig. 5).

We also compared the relative timing of the onset of ossification in the fat-tailed dunnart to two other Dasyuridae species, *Dasyurus viverrinus* (Eastern quoll)^13,80^ and *Sminthopsis macroura* (the stripe-faced dunnart), as well as the reconstructed Marsupialia ancestor^64^. *S. crassicaudata* (this study) similarly had early ossification of the maxilla, premaxilla and dentary but greater variation in onset of ossification for other bones of the skull with the exception of the petrosal bone (Supplementary Fig. 5b). Aside from the petrosal bone, there was no overlap in the timing of the onset of ossification in the other cranial bones with another dunnart species, the striped-faced dunnart (*S. macroura*).

### Onset of cranial bone contacts

**Fig. 6:**
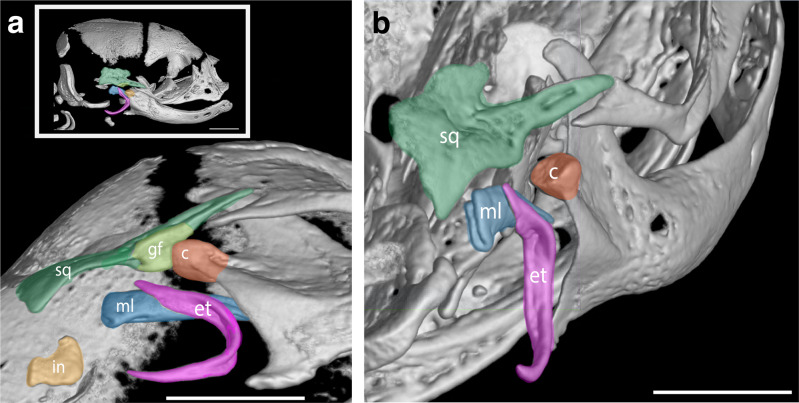
The secondary jaw joint as revealed in microCT scans in D20 fat-tailed dunnart pouch young. **a** Ventral view of the secondary jaw joint with inset white box showing entire skull. **b** Posterior to anterior view of the secondary jaw joint. c = condyle (orange), et = ectotympanic ring (purple), gf = glenoid fossa (light green), in = incus (yellow), ml = malleus (blue) and sq = squamosal (dark green). Scale bar = 1 mm.

**Fig. 7:**
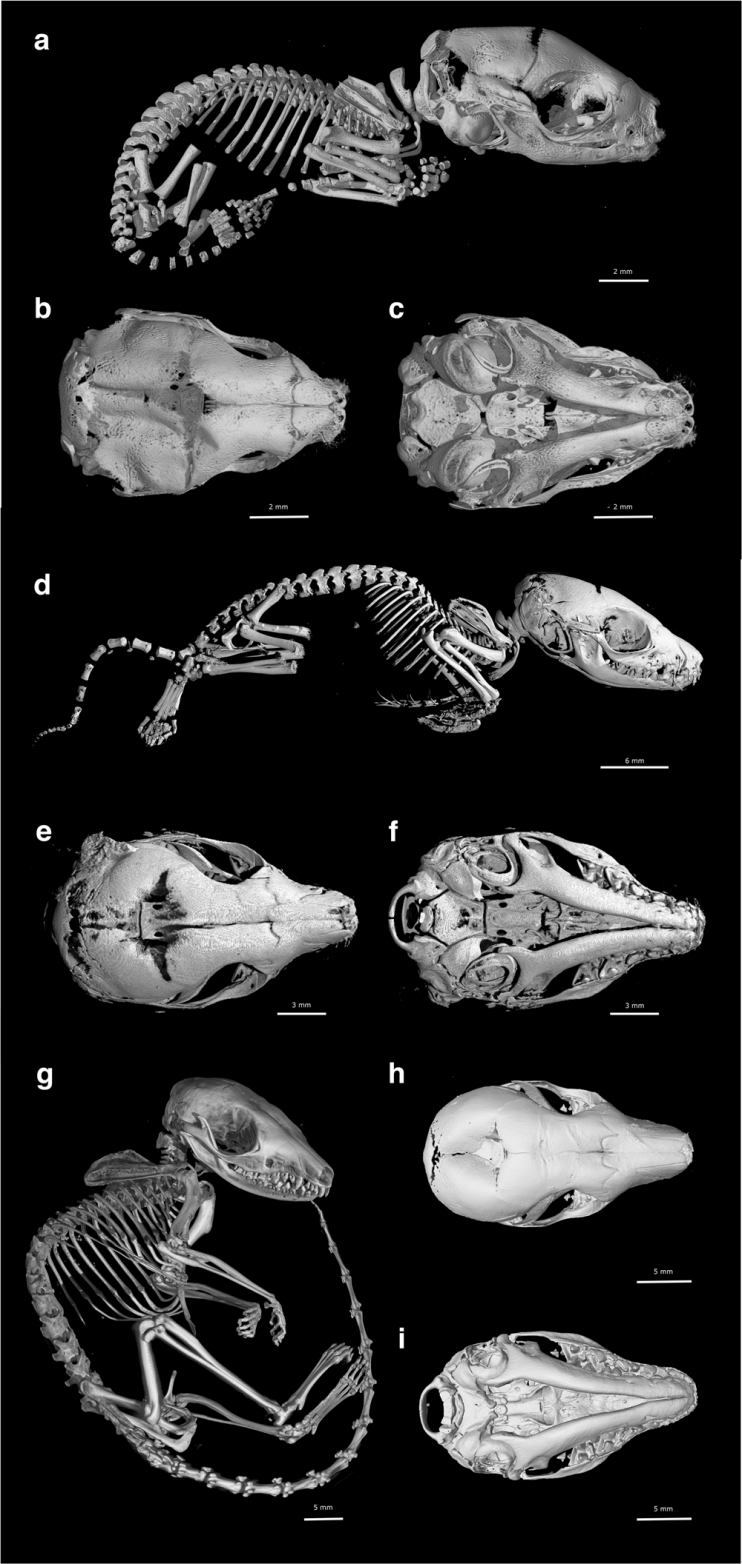
The skeleton of fat-tailed dunnart pouch young (D35, D50 and D70), as revealed in microCT scans. The right lateral side of the skeleton is shown in (**a**), (**d**) and (**g**), the dorsal view of the skull in (**b**), (**e**) and (**h**), and the ventral view of the skull in (**c**), (**f**) and (**i**). Pouch young on D35 are shown in (**a**), (**b**) and (**c**). Pouch young on D50 are shown in (**d**), (**e**) and (**f**). Pouch young on D70 are shown in (**g**), (**h**) and (**i**).

The bones surrounding the oral cavity connected early relative to the bones of the cranium, with the maxilla-palatine bone contact event on D8, lacrimal-maxilla and premaxilla-maxilla on D10, premaxilla-nasal on D15, and maxilla-nasal on D25. On D20 the secondary jaw joint (temporomandibular joint) is present with a mortar-shaped condylar head and a concave-shaped glenoid fossa (Fig. 6a, b). Other early connecting bones include the goniale-ectotympanoid (D5) and goniale-malleus (D10) (Supplementary Figs. 6 and 7). On D30, bone contacts in the cranium had begun with the supraoccipital-interparietal, parietal-frontal, frontal-nasal, frontal-lacrimal and alisphenoid-frontal (Fig. 7a–c; Supplementary Fig. 6). The latest bone contacts observed in this cranial series were the supraoccipital-petrosal, supraoccipital-squamosal, and basioccipital-basisphenoid which had just begun to make contact at weaning (D70; Fig. 7g–i, Supplementary Fig. 6). The onset of bone-contacts observed in the fat-tailed dunnart showed a similar pattern to both the Eastern quoll^13,80^ and the reconstructed Marsupialia ancestor^64^ with the exception of the supraoccipital-squamosal bone contact (relative timing Marsupialia = 0.17 and *S. crassicaudata* = 1.0), maxilla-jugal (Marsupialia = 0.20, *D. viverrinus* = 0.14, dunnart = 0.67), supraoccipital-interparietal (Marsupialia = 0.29, *D. viverrinus* = 0.14, *S. crassicaudata* = 0.67), jugal-squamosal (Marsupialia = 0.39, *D. viverrinus* = 0.50, *S. crassicaudata* = 0.78; Supplementary Fig. 5a).

### Postcranial ossification

On D1, ossification centres of thoracic and cervical vertebrae were present either side of the neural arch (Fig. 4a). The atlas had begun to ossify by D2 and ossification centres of the lumbar vertebrae were present on D3. The caudal vertebrae did not begin ossification until two days later on D5, coinciding with the onset of ossification of the first bone of the pelvic girdle, the ilium (Fig. 4d). The ischium ossifies later and was evident on D15 (Fig. 4g). The ossification centre of the distal section of the epipubic bone was present on D20. Ossification centres were present in the scapula, clavicle and the body of the first six rib pairs on D1 (Fig. 4a). By D4 the body of the true, false and floating ribs were all undergoing ossification. The capitulum of each rib had an ossification centre on D7. The first ossification centres of the sternum were evident on D15 in the xiphoid process, manubrium and the first three anterior sternebrae of the body (Fig. 4g). The fourth sternebrae began ossification later and was evident on D25.

Cartilage was present in the forelimb but there was no cartilage present in the hindlimb on the day of birth or D1 (Fig. 3c, d). One day after birth, ossification centres in the humerus, radius and ulna were evident (Fig. 4a; Table 2). On D5 the bones of the hindlimbs began to ossify, with the femur, tibia and fibula present (Fig. 4a). Ossification centres were present on D6 in the forelimb phalanges but not until D15 in the hindlimb phalanges (Fig. 4d; Table 2). The metacarpals of the forelimbs had begun ossification on D10 and the metatarsals of the hindlimbs by D15 (Fig. 4g; Table 2). Ossification centres of the carpals and tarsals were the last to ossify and were evident on D40 (Fig. 7d; Table 2).

**Table 2:** Onset of ossification centres observed in the cranial and postcranial skeleton of *S. crassicaudata* pouch young.

Age (days post birth)	Cranial	Post-cranial
0	–	–
1	Premaxilla, maxilla, dentary, exoccipital, palatine process	Scapula, clavicle, humerus, ulna, radius, ribs, thoracic and cervical vertebrae
2		Atlas
3	Pterygoid, basioccipital	Lumbar vertebrae
4	Jugal, basisphenoid, frontal	
5	Supraoccipital, squamosal, nasal, lacrimal, ectotympanic, parietal, goniale	Caudal vertebrae, femur, tibia, fibula, ilium
6	Alisphenoid	Forelimb phalanges
7	Malleus	
10		Metacarpals
15	Incus, interparietal	Hindlimb phalanges, sternum, ischium, metatarsals
20	Petrosal	Epipubic
40		Carpals, tarsals

### Allometric growth patterns during fat-tailed dunnart ontogeny

**Fig. 8:**
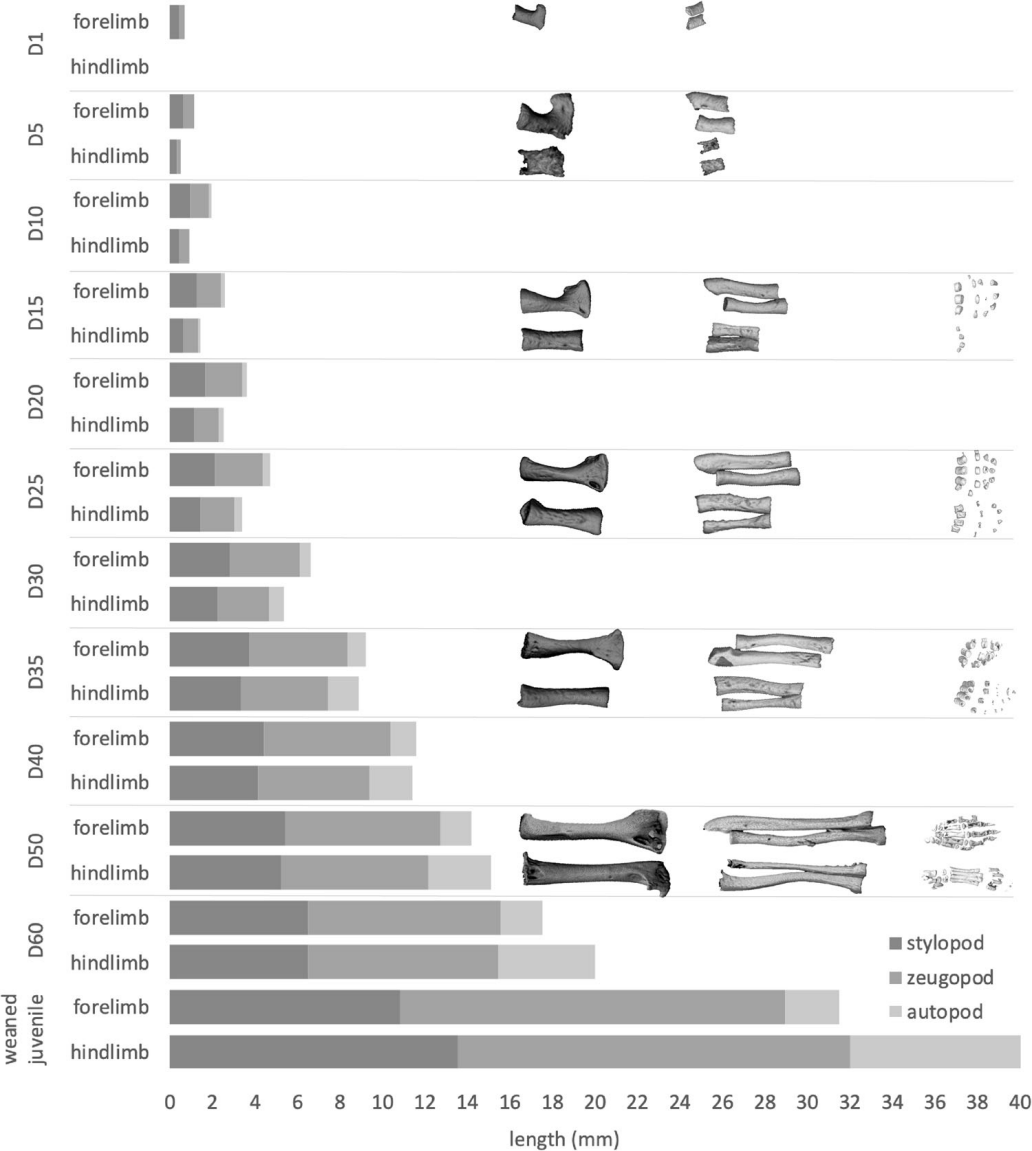
Limb heterochrony in the fat-tailed dunnart. Relative lengths of forelimb and hindlimb elements throughout development, shown as shaded grey bars (*n* = 1). Limbs are divided into proximal and distal segments: the stylopod (humerus and femur), zeugopod (ulna/radius and tibia/fibula) and autopod (carpals, metacarpals, tarsal, metatarsals and digits).

Pouch young display heterochrony of the limb bones from day one to approximately day 50 (Fig. 8). Individual bones of the dunnart limbs also displayed pronounced differences in allometric scaling during growth (Fig. 8). Following birth, pouch young possess well-developed forelimbs, showing ossification of the humerus, radius and tibia, but possess underdeveloped, paddle-like hindlimbs lacking any bone. By D5, the hindlimbs show their first signs of extension and development, emphasizing the strong developmental heterochrony. The autopod of the forelimb (carpus) and hindlimb (tarsus) are roughly the same length from D15 to D30 (Fig. 8). After D30 the autopod of the hindlimb overtakes the carpus and this difference in length continues to expand until the weaned juvenile stage.

## Discussion

Here, we present a high-resolution, digital reconstruction of the complete postnatal ontogeny of *S. crassicaudata*. Our study focused on characterizing early skeletal development in the fat-tailed dunnart to establish it as a comparative laboratory-based model for mammalian skeletal osteogenesis. Our description of postnatal gross morphology including head length, weight and crown-to-rump length provides a staging tool for future studies using this marsupial model. This expands upon a previous staging series^54^ to include detailed skeletal descriptions and a general overview of some interesting aspects of organ development.

### The fat-tailed dunnart at birth

Newborn dunnarts have large heads (almost 50% of the total body length) and well-developed forelimbs compared to hindlimbs that are required to facilitate movement to the pouch and teat attachment. They lack an ossified skeleton, showing a cartilaginous postcranial skeleton and chondrocranium. The chondrocranium is a transitory, embryonic structure critical in the developing mammalian head^81,82^. In fat-tailed dunnarts, the newborn young display large medial nasal swellings. This is consistent with descriptions of the chondrocranium in *Sminthopsis virginiae* (the red-cheeked dunnart), which has the most extensive cartilago cupularis (the rostral part of the nasal cartilage) of any marsupial examined^83^. Previous studies have shown that variation in the *cupula nasi* anterior (the anterior portion of the cartilaginous nasal capsule) reflects the animal’s life history^83^. The nasal capsule morphology in marsupials is more uniform across the group than observed in placentals and this is thought to be related to the extended fixation of pouch young to the mother’s teat during lactation^83^. It has also been proposed that there is a relationship between the innervation of the area proximal to the *cupla nasi* anterior and the sensory requirements of marsupials at birth^84–86^. An in vitro study in *M. domestica* newborn young found that when pressure was applied to the innervated snout this induced electromyographic responses from the triceps muscle in both forelimbs^87^. The forelimb responses were absent when the facial skin was removed, suggesting that the journey to the pouch may be aided by the influence of facial mechanosensation on forelimb movement^87^. Dunnart neonates lack a distinct neck and any skeletal elements to support the head and the journey to the pouch is instead aided by a large cervical swelling which supports the head until D4. The cervical swelling is also thought to assist with teat attachment^88^. Cervical swellings have been reported in two other Dasyuridae species: the Tasmanian devil, *Sarcophilus harrisii* and Eastern quoll, *Dasyurus viverrinus*^8,88^ and are correlated with the altricial nature of the pouch young. Marsupials with more precocious newborns, such as macropods (e.g., kangaroos, wallabies), lack cervical swellings and have full head movement at birth^8,33^.

### Internal organ development

The microCT scans provide an excellent resource for studying the development of internal organs, particularly in the older stages where the scans are clearer. We have briefly described aspects of lung, gonad and kidney development but make these data publicly available for additional studies of organ development in more detail. The lungs of dunnart newborns were characteristic of primitive airways with large tubular-like structures. On the day of birth, dunnart lungs are similar to that of eutherian lungs during the canicular stage of embryonic development^89^. At birth, in the fat-tailed dunnart, the skin is responsible for almost all gas exchange with pulmonary ventilation unable to satisfy the demand for oxygen until between D23 and D35^89^. Interestingly, this is when we observe the rapid increase in lung complexity between D30 and D35. It has been suggested that the short gestation time of dunnarts provides insufficient time for development of a complex respiratory system and therefore relies on transcutaneous gas exchange^89^.

Remarkably, we were able to capture the process of testicular descent through the inguinal canal into the scrotum. On D40, the testes had migrated through the abdomen and were ready to begin the inguinoscrotal phase of testicular descent. At D50, the testes were visible at the neck of the scrotum and by D60 were situated in the base of the scrotum. It has previously been suggested that the time taken for completion of testicular descent in marsupials may be associated with postnatal growth rate^90^ given the shorter period (3–4 weeks) for testicular descent in the bandicoot (*Perameles gunni*)^91^ compared to kangaroos, wallabies and possums (10–11 weeks)^74,92–94^. However, in our study we find that although dunnarts represent one of the most altricial neonates (Grade 1^8^), the process of testicular descent occurs at almost exactly the same postnatal stage as in other more precocial marsupials^74,92–94^. Our results are also supported by a study in *Antechinus stuartii* (family Dasyuridae), which found that after one month the testes were situated in the inguinal canal and after two months were present at the base on the scrotum^95^.

### Craniofacial development

Using a combination of microCT scanning and histological techniques we confirmed that on the day of birth (<24-h old), no ossified bones were present in the dunnart. Mineralized bone was observed one day after birth and was limited to bones in the oral region, forelimbs and vertebrae. We observed osteoid matrix in the maxillary and dentary of D0 pouch young head sections showing that ossification had begun. This suggests that microCT is useful for assessing when ossified bone arises, however, histology is still required for investigating the onset of ossification prior to mineralization.

Previous studies of skeletal development in marsupials have similarly shown that the maxilla, premaxilla and dentary are the first bones to ossify^59,61–64^. However, there appears to be some differences in the timing of onset of ossification in different marsupials. In *Monodelphis domestica* and *Macropus eugenii* these bones have already begun ossification prior to birth^59^. In contrast, in *Isoodon macrourus*^62^, *Trichosurus vulpecula*^62^, *Didelphis albiventris*^60^ and *Sminthopsis macroura*^61^ ossification begins after birth. Although marsupials show inter- and intraspecies variation in the timing of the onset of ossification in bones of the skull, the relative timing of bone contacts in the oral region, middle ear and late occipital region are highly conserved^64^. Similar to Spiekman and Werneburg^64^, we observed the first bone contacts occurred in bones surrounding the mouth cavity (maxilla-palatine) and middle ear bones (ectotympanic-goniale and goniale-malleus). In mammals the middle ear bones form as part of the mandible and separate after the secondary jaw joint has formed^48,96^. However, due to the altriciality of newborns, marsupials and monotremes are born before the jaw joint forms and use their middle ear bones to articulate the lower jaw with the head to allow for feeding^82,97–100^. In the dunnart the secondary jaw joint is formed by D20, which is comparable with the opossum (D14–D20^82,96,101^). The last bones to make contact were the those that form the back of the cranium, particularly in those that connect the occipital bones. As previously observed in marsupials^64^, the connections within the occipital region occurred around the time of weaning, suggesting that the region is not required for cervical support during suckling^64^.

Developmental comparisons to the well-known placental model, the laboratory mouse (*Mus musculus*), are useful for studying the evolution of skeletal elements, particularly given the heterochrony with in marsupials. Most ossification centres in placentals are present at birth, with ossification in mice beginning on embryonic days 12–13^102–104^, while in the dunnart all ossification occurs postnatally. Similar to the dunnart, the bones of the oral region (premaxilla, maxilla, dentary, palatine and pterygoid) are the first to ossify in the mouse, presumably in preparation for feeding, as in marsupials. However, unlike in the dunnart and other marsupials, the mouse cranium (basioccipital, frontal, parietal and sphenoid) ossifies at a similar stage of development as the oral bones. The developmental timing of the facial morphology appears to be less disparate across marsupials than placental mammals, but displays equal variation in the neurocranial morphology^105^. This difference in cranial developmental timing between marsupials and placentals is thought to be driven by the early functional requirements of the oral apparatus for continuous suckling in marsupials^105^. However, the bones of the oral regions (premaxilla, maxilla, dentary and palatine) are also the first to ossify in the reconstructed ancestral ossification sequence of Mammalia^63^ and often in non-mammalian amniotes suggesting prioritized development of the oral apparatus is the ancestral state in amniotes^63,64,106–109^. The short gestation time and altricial stage of the marsupial neonate at birth is likely to have driven a more extreme shift in the developmental timing of this event, relative to the rest of the body. This is supported by the extremely high integration of the oral bones in early marsupial postnatal ontogeny in comparison to other cranial regions where levels of integration are similar to placentals^17^. In particular, cranial bones arising from the migratory neural crest cell population of the first pharyngeal arch have been shown to be constrained between marsupials to facilitate early functional demands^66^.

Early ossification of the craniofacial bones in *Monodelphis domestica* (grey short-tailed opossum) is thought to occur through accelerated migration, proliferation and differentiation of the cranial neural crest cells that pattern the facial prominences and skeletal elements^110,111^. A marsupial-specific region within a *Sox9* enhancer was found to drive early and broad expression of *Sox9* in pre-migratory neural crest cell domains contributing to early migration of cranial neural crest cells relative to the mouse^112,113^. The spatio-temporal expression of downstream key ossification genes such as *Runx2* and *Osx* have not been studied and in comparison to placentals, little is known of the molecular and genetic control of craniofacial development in marsupials^96,97,114^. Recently, Smith (2020) presented previously unpublished work of J.P. Hill and Katherine Watson on the development of neural crest in other marsupial taxa^115^. They confirmed that early migration of the neural crest occurs in multiple marsupial taxa, particularly for the neural crest cells that end up in the facial and first arch regions^115^. Interestingly, dasyurids differed from other marsupials with considerable accumulation of neural crest before somitogenesis had begun, along with far earlier migration of the ectomesenchyme into the craniofacial region^115^. Smith (2020) suggested that the shorter the period of gestation in marsupials the earlier the accumulation and migration of neural crest will be. Being one of the most altricial marsupials in existence, the dunnart provides an ideal model in which to study the molecular drivers of heterochrony in craniofacial ossification in marsupials.

Based on the facial processes present in the newborn dunnart and the lack of ossification centres, we propose that at D0 dunnart craniofacial development corresponds to that of the E11.5–E12 mouse embryo. This suggests that at birth in the dunnart, neural crest cells are still proliferating and differentiating. Given this new developmental information, the dunnart therefore presents a new and exciting mammalian model to expand craniofacial research as facial primordia can be manipulated *ex utero* and the role of key genes underlying osteogenesis can be directly observed.

### Limb development

The ossification sequence of the dunnart postcranial skeleton follows a similar pattern to that previously described in marsupials^59,61,62,65^. Like all marsupials, dunnart pouch young display heterochrony in the development of the limb bones with the forelimbs being longer than the hindlimbs from D1 to ~D50. After D50, the limbs undergo a heterochronic shift in their length and the hindlimbs overtake the length of the forelimbs. This corresponds with the pouch young no longer being permanently attached to the teat in the lead up to weaning^58^. The bones of the carpals and tarsals were the last to ossify in the limbs. The ossification of the metacarpals, metatarsals and digits before the carpals and tarsals is consistent with ossification patterns in *S. macroura*^61^, *Trichosurus vulpecula*^62^, *Thylacinus cynocephalus*^65^ and *Didelphis alviventris*^60^. Embryological and molecular studies suggest that marsupial limb heterochrony is driven by both an acceleration in the development of the forelimb buds and a delay in the development of the hindlimb buds^7,9,10,116–118^. In addition to the heterochrony between the fore- and hindlimbs in marsupials, heterochrony is also observed in the timing of the onset of marsupial forelimb and hindlimb development relative to placentals. Dunnart forelimb development is accelerated compared to the hindlimb, and compared to that in the mouse^119^, where both limb buds begin to differentiate and grow at a similar stage of development^119,120^. On the day of birth, the dunnart forelimb has well-developed musculature, and digits with claws to facilitate its crawl to pouch. However, the D0 dunnart lacks ossified forelimb bones, suggesting that it does not require fully formed skeletal elements to complete the crawl to the mother’s pouch. Conversely, the hindlimb has no cartilage or bone and is a paddle-like bud with digital condensations beginning to form. Generally, mammalian forelimbs develop ahead of hindlimbs, with the timing relatively close in placentals, while in marsupials the difference in the rate of forelimb and hindlimb development is extreme^6,121^. The dunnart represents one of the most altricial neonates^8^, and so is an extreme example of this heterochronic shift in forelimb and hindlimb development. The heterochrony in marsupial limb development involves the early specification and initiation of patterning in the forelimb bud relative to foetal development^122^. In the opossum (*M. domestica*), the early initiation of the forelimb field is initiated during the early stages of somitogenesis through accelerated expression of the necessary forelimb transcription factor *TBX5*, and limb outgrowth and patterning genes *FGF10*, *FGF8* and *SHH*, and greater myocyte (forming the later limb musculature) allocation^42,122^. *SHH* is also expressed early in the forelimbs of tammar wallaby (*Macropus eugenii*) embryos relative to hindlimb bud expression^123^.

Research into marsupial limb development is currently limited by the inability to perform in vivo transgenic experiments^42,44,122–127^. Although limb organ cultures have been successfully used in the opossum to investigate the molecular control of limb heterochrony^124^, limb development has predominantly been studied in the chick and mouse given the ease of observing and manipulating limb patterning in these species. Electroporation technologies previously used in chick^128^, xenopus^129^ and mice^130^ have recently been successfully applied to manipulating dunnart brain development^55^. This technology provides a tangible system for the manipulation of gene expression in the fat-tailed dunnart in vivo.

### Future directions

The public availability of precisely staged microCT scans for the fat-tailed dunnart as presented here provides a valuable resource for future studies in mammalian development. The highly altricial state of the newborn dunnart allows for precise, tissue-specific manipulation of skeletal development *ex utero*, prior to the onset of ossification and over an extended period of time. Such manipulations are challenging to perform in placental mammals where ossification is established *in utero*. The dunnart provides access to key ossification stages in an easily manipulable mammalian model. A staging series of the fat-tailed dunnart therefore presents a fundamental resource that will underpin future work into marsupial development, in particular defining the molecular control of craniofacial and limb heterochrony.

## Methods

### Collection of pouch young

All animal procedures, including laboratory breeding, were conducted in accordance with the current Australian Code for the Care and Use of Animals for Scientific Purposes^131^ and were approved by The University of Melbourne Animal Ethics Committee (AEC: 1513686.2) and with the appropriate Wildlife Permit (number 10008652) from the Department of Environment, Land, Water and Planning. Animals were housed in a breeding colony in the School of BioSciences, The University of Melbourne. Animals are bred using the Poiley outbreeding system to limit inbreeding^132^ and breeding boxes were set up with a male:female ratio of 1:2 or 1:3. Animals were kept in cages with water and vitamin supplements (Pentavite; 1 mL into 100 mL water) changed three times a week. All cages had nest boxes with shredded newspaper, empty toilet rolls, drinking bottle, food bowl and native tree branches. Animals were fed each day a diet consisting of live food (2 crickets and 3 mealworms) and wet mince mixture of 51% beef mince (Whiskas), 36% beef and lamb flavoured biscuits (Whiskas), 12.7% wombaroo and 0.3% calcium carbonate. Dunnart were kept on a 16:8-h light:dark cycle and temperature between 21 and 25 ^∘^C. Females were monitored to track oestrus cycle^133^. Body weight fluctuations in female animals and daily examination of urine samples under light microscopy were used to determine whether ovulation had occurred. A decrease in body weight and the presence of cornified epithelial cells in the urine is associated with the day of ovulation^133^. The presence of sperm in the urine of females confirmed that insemination had occurred. Pregnancy is timed with the detection of ovulation or the appearance of sperm set as day 0 of pregnancy^133^. Female pouches were checked every day for births by gently holding the animal with one hand and opening the pouch with the thumb and index finger of the other hand. On the day of birth, the mothers often have a small amount of blood in the urine and the pouch young will be pink and highly vascularised. Pouch young were removed by gently holding the female on her back with one hand and using the thumb and index finger of the other hand to gently but quickly slide the young from the teat. Pouch young under D15 were placed on ice for 15 min as an anaesthetic followed by immersion-fixation in 4% paraformaldehyde (PFA) in phosphate-buffered saline (PBS). Pouch young from D15 to D45 were anaesthetised on ice for 15 min, followed by intraperitoneal (IP) injections of 4% PFA in PBS. Pouch young D50 and older were anaesthetised via an intramuscular injection of 10 mg kg^−1^ Zoletil (1:1 tiletamine HCl, zolazepam HCl), then killed with an 0.05–0.1 mL volume intraperitoneal injection of 150 mg kg^−1^ pentobarbital sodium (made up to 60 mg ml^−1^ in sterile saline) After fixation specimens were washed twice with PBS for 1 h and then dehydrated in increasing ethanol solutions before storing in 70% ethanol. Pouch young were photographed using a Nikon Digital Sight DS-U3 (Nikon, Tokyo, Japan) and all images were processed through NIS Elements Analysis D v.4.300.00 64-bit software (Nikon).

### MicroCT scanning and skeletal reconstructions

Specimens were collected every day from D0 to D10, then every 5 days from D10 to D50, then every 10 days from D50 to D70. Specimens were scanned using X-ray micro-computed tomography (microCT) at the TrACEES platform, School of Earth Sciences, University of Melbourne. One specimen from each stage was stained with 1% iodine in 100% ethanol in order to visualise soft tissue in the microCT images. Specimens under D40 were stained for 24 h and specimens over D40 were stained for 72 h. Specimens were rinsed in 100% EtOH once before scanning. Specimens were either mounted in 200–1000 μL pipette tips (Axygen) suspended in ethanol or suspended dry with cotton wool spacers between specimens. Larger specimens were wrapped in standard bubble wrap and scanned in either a 15 or 50 mL Falcon tube (Sigma). MicroCT scanning was performed in a Phoenix Nanotom m (GE Sensing & Inspection Technologies GmbH) operated using xs control and Phoenix datos—x acquisition and reconstruction software (GE Sensing & Inspection Technologies). Samples were scanned for 10 minutes at varying resolutions ranging from 4.75 to 26.18 μm pixel size (Supplementary Data 1). The X-ray energy varied from 40 to 55 kV and 300 to 250 μA depending upon the size of the specimen and whether it was stained with iodine (Supplementary Data 1). A Diamond target was used and 1199 2D X-ray projections were collected over a 360° rotation of the specimens. Skeletal reconstructions were performed in VGStudio MAX 3.0 (Volume Graphics, Heidelberg, Germany). Left and right-side limb bones were measured for one individual per sampled age and averaged using polyline length tool in VGStudio MAX 3.0 (Volume Graphics, Heidelberg, Germany). First appearance of skeletal elements and cranial bone contacts were observed in VGStudio MAX 3.0 for one specimen from each precisely staged age and recorded (Volume Graphics, Heidelberg, Germany). To compare to previously published datasets from other mammalian species, specimens were ranked in order of bone onset timing or bone contact timing, and relative ranks were distributed between 0 and 1 as described in Koyabu et al.^63^. Original and relative ranks are listed in Supplementary Table 1 and Supplementary Data 2 and 3.

### Histology

Pouch young were collected (see the ‘Collection of pouch young’ section) on D0 and D1 either fixed in 4% paraformaldehyde for serial sectioning or fixed in 95% ethanol for whole-mount staining. Samples for serial sectioning were processed through a series of ethanol and xylene washes (15-min steps; Tissue-Tek VIP, School of BioSciences), embedded into paraffin wax (Leica) and 7-μm sections cut with a microtome (Zeiss, Sydney, Australia) and transferred to superfrost slides (Platinum Pro, Grale). For D0 (*n* = 1) and D1 (*n* = 1), sections were either stained with Harris’ Haematoxylin (Australian Biostain) and Eosin Y (Australian Biostain), Alizarin Red (ProSciTech, Australia) or Alician Blue (ProSciTech, Australia) according to standard methods^134^, or a modified Masson’s trichrome stain^135,136^. Sections were imaged on an Olympus BX51 Microscope with an Olympus DP70 Camera (Olympus, Sydney, Australia). Bone and cartilage wholemount staining was performed according to the described protocol for mouse embryos^137^. Briefly, pouch young skin (D0, *n* = 2; D1, *n* = 2) was removed with forceps, stained in 0.05% alcian blue stain solution overnight then washed for 8 h in 70% ethanol. Specimens were cleared with 1% potassium hydroxide for 2 h and counterstained with alizarin red stain solution (0.005% (w/v) alizarin red in 1% potassium hydroxide) overnight. Specimens were cleared in 1% potassium hydroxide/20% glycerol for 2 days and stored in glycerol:ethanol (1:1). Stained pouch young were photographed using a Nikon Digital Sight DS-U3 (Nikon, Tokyo, Japan) and all images were processed through NIS Elements Analysis D v.4.300.00 64-bit software (Nikon).

### Reporting summary

Further information on research design is available in the Nature Research Reporting Summary linked to this article.

## Supplementary information

Supplementary Information

Description of Supplementary Files

Supplementary Movie 1

Supplementary Movie 2

Supplementary Movie 3

Supplementary Movie 4

Supplementary Movie 5

Supplementary Movie 6

Supplementary Movie 7

Supplementary Data 1

Supplementary Data 2

Supplementary Data 3

Reporting Summary

## Data Availability

All X-ray and reconstructed microCT data shown are publicly available through a MorphoSource repository (www.morphosource.org, Project P1150)^138^. Histology slide images are available through a Figshare repository (www.figshare.com, Project 111930)^139^. For comparison of ossification patterns between species, timing and ranks were obtained from two previous publication datasets by Koyabu et al.^63^ and Spiekman et al.^64^.
