# Supplementary material for: Postnatal development in a marsupial model, the fat-tailed dunnart (Sminthopsis crassicaudata; Dasyuromorphia: Dasyuridae)
Source: Commun Biol. 2021 Sep 2;4:1028. doi: 10.1038/s42003-021-02506-2 (PMC8413461; doi:10.1038/s42003-021-02506-2)
Supplement: Supplementary file 1 — Supplementary Information [file 42003_2021_2506_MOESM1_ESM.pdf]

Supplementary Information for:

Postnatal development in a marsupial model, the fat-tailed dunnart (*Sminthopsis crassicaudata*; Dasyuromorphia: Dasyuridae)

Laura E. Cook[1], Axel H. Newton[2], Christy A Hipsley[1,3,4], Andrew J. Pask[1,4]*

1 School of Biosciences, University of Melbourne, Royal Parade, Parkville, Victoria, Australia

2 Department of Anatomy and Developmental Biology, Monash University, Clayton, Victoria, Australia

3 Department of Biology, University of Copenhagen, Copenhagen, Australia

4 Department of Sciences, Museums Victoria, Carlton, Victoria, Australia

* To whom correspondence should be addressed: ajpask@unimelb.edu.au

Supplementary Figures

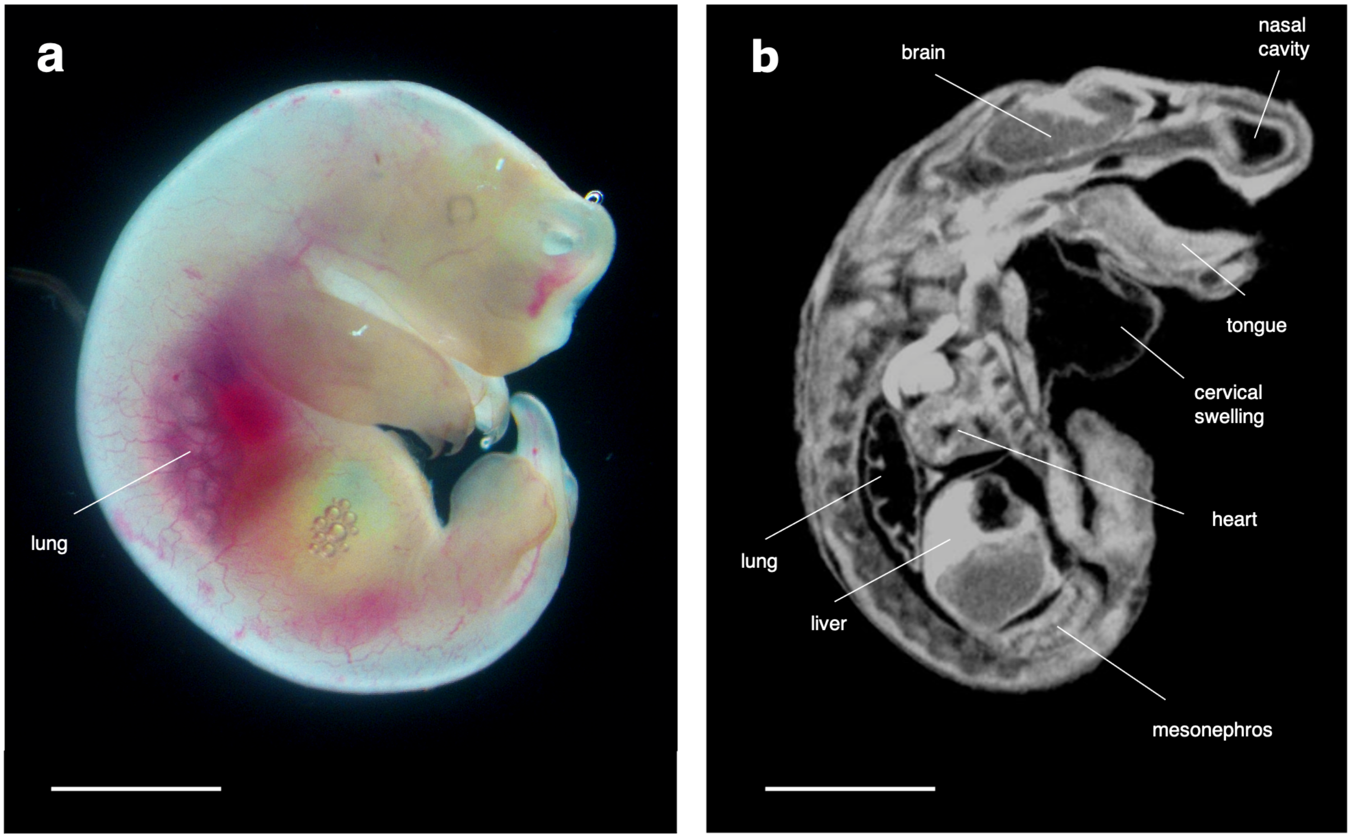

Supplementary Figure 1. Fat-tailed dunnart on the day of birth. **a** Right lateral wholemount image. **b** Sagittal slice from microCT scan highlighting internal organs. Scale bar = 1mm.

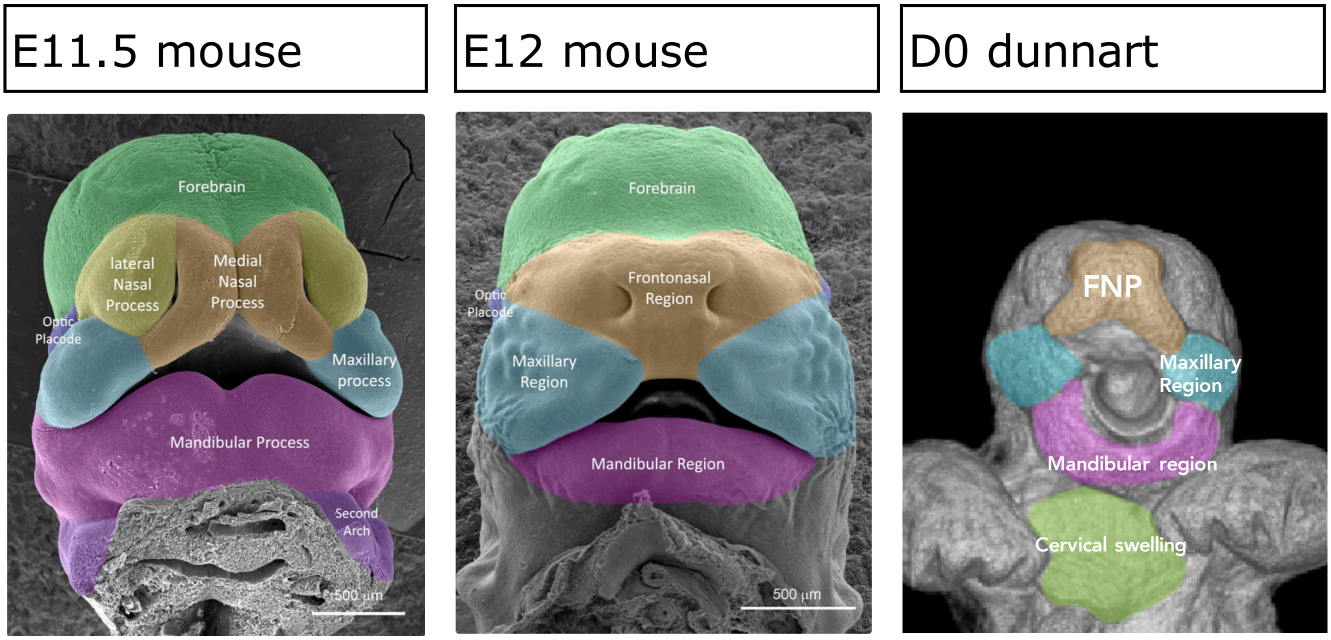

Supplementary Figure 2. Color-coded scanning electron microscopy images for C57BL/6 mouse embryo craniofacial anatomy at embryonic stage (E) E11.5 and E12 [1, 2] alongside microCT surface reconstruction of D0 *S. crassicaudata*. FNP = frontonasal process.

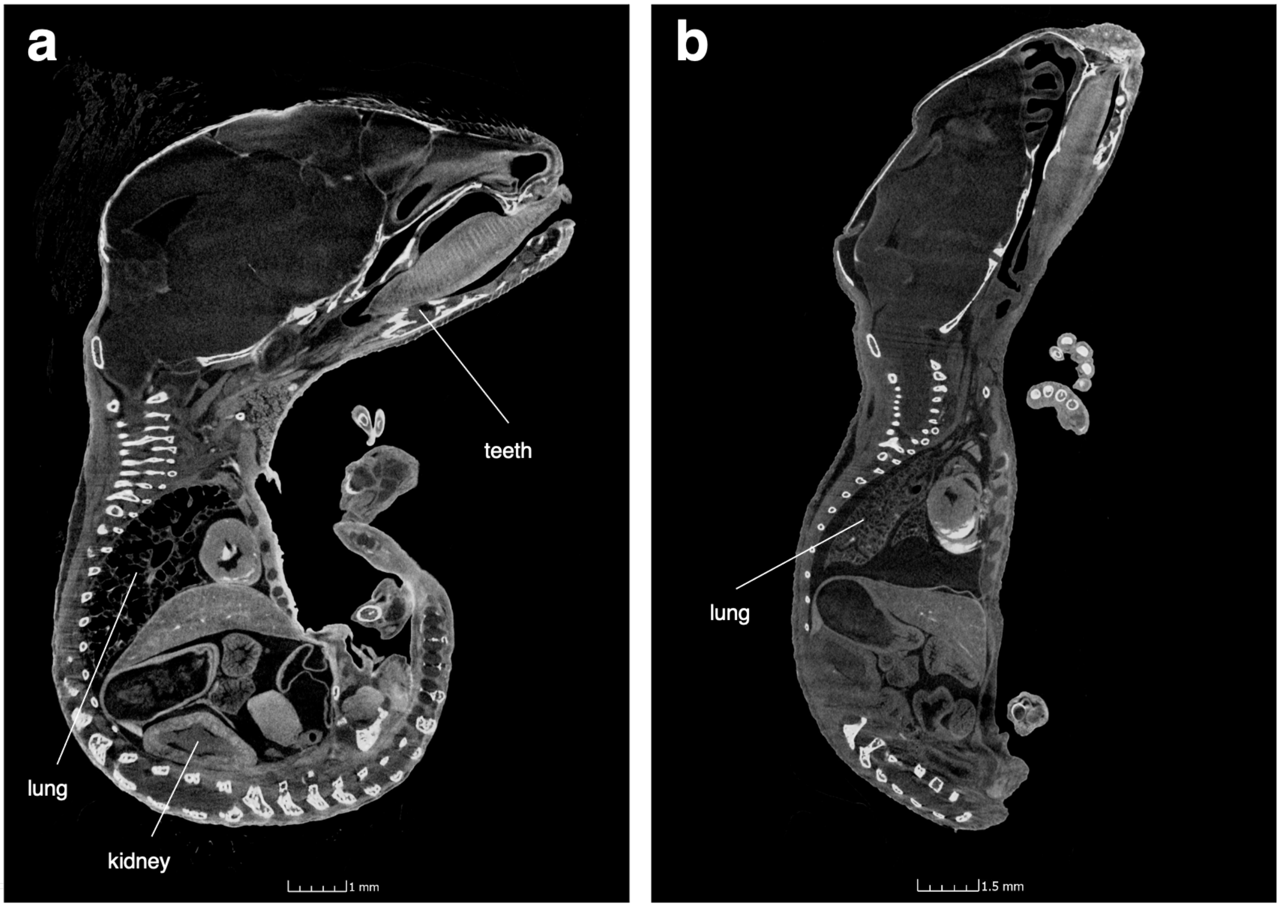

Supplementary Figure 3. Sagittal slice from a microCT volume rendering of the fat-tailed dunnart **a** D30, and **b** D35.

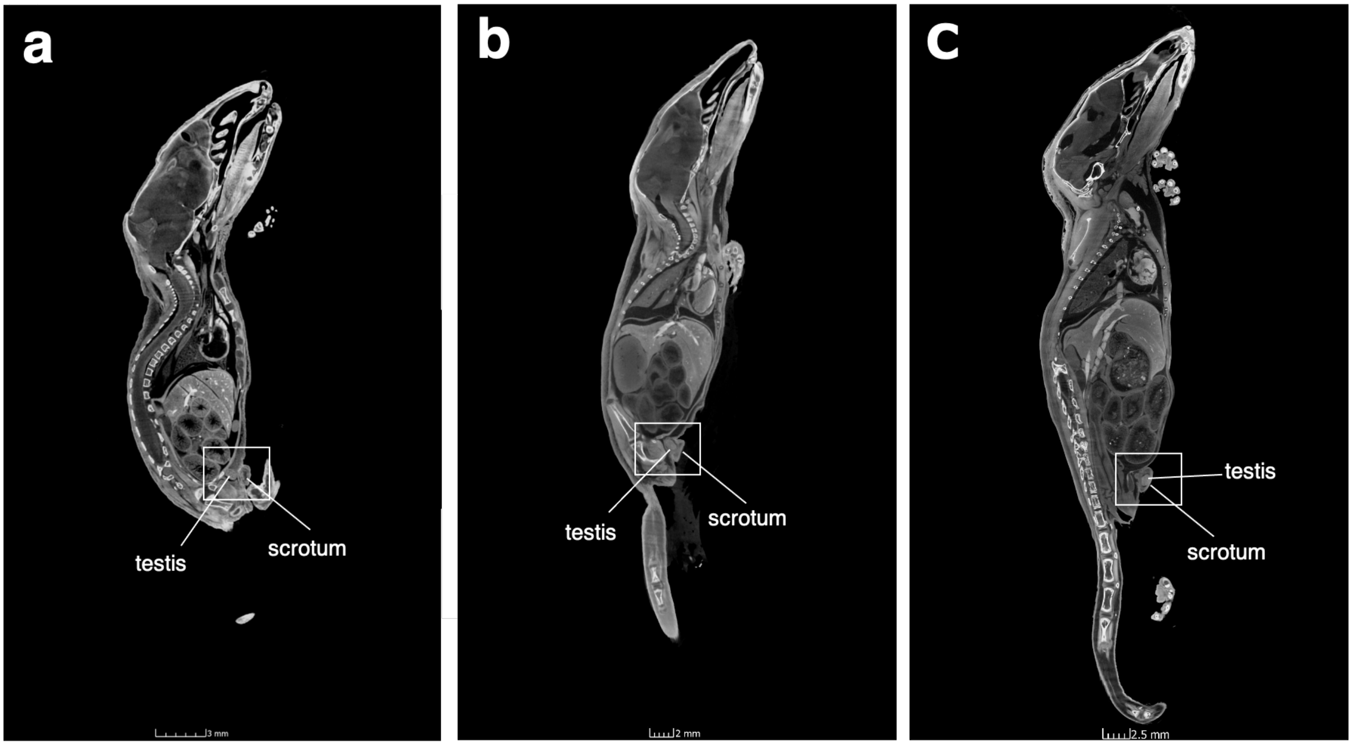

Supplementary Figure 4. Sagittal slice from a microCT volume rendering of the fat-tailed dunnart **a** D40, **b** D50, and **c** D60.

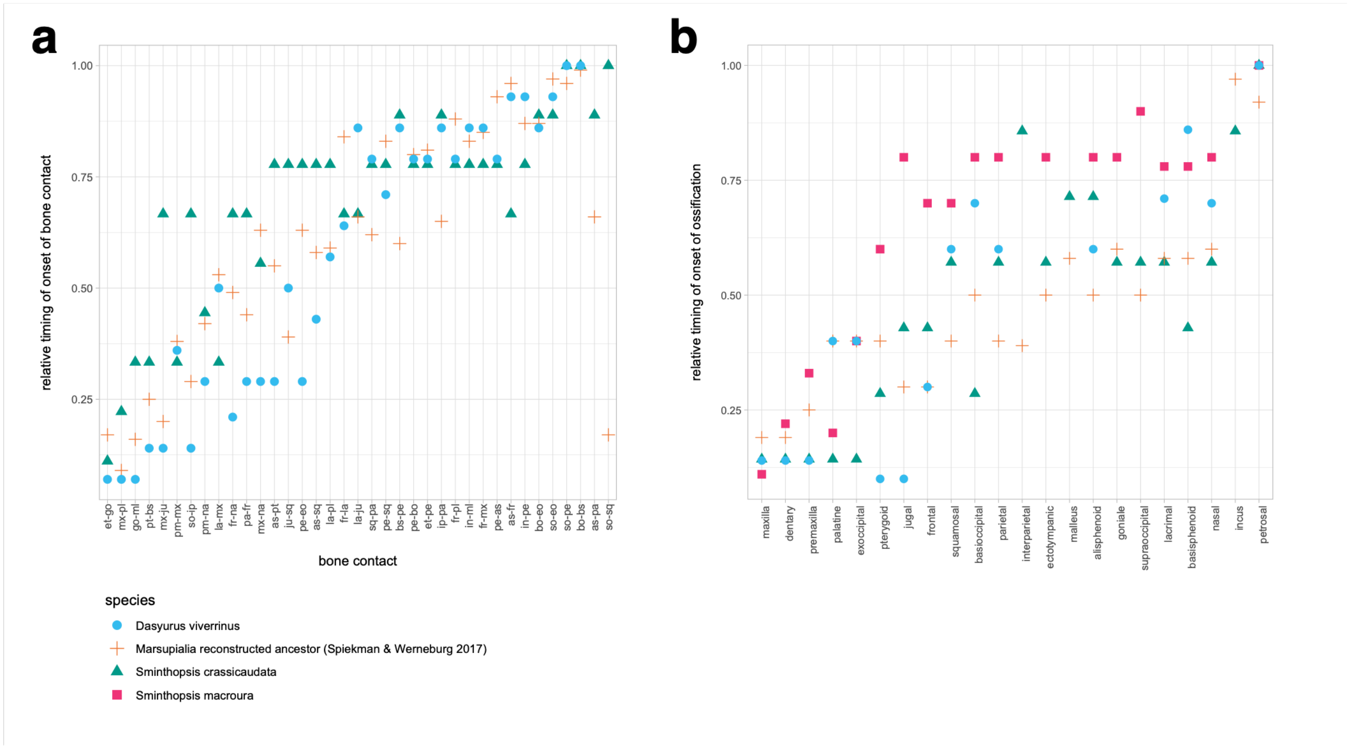

Supplementary Figure 5. **a** Relative timing of onset of bone contacts and **a** onset of ossification for *S. crassicaudata* (this study), *D. viverrinus* [3, 4] and the reconstructed ancestor of Marsupialia [5].

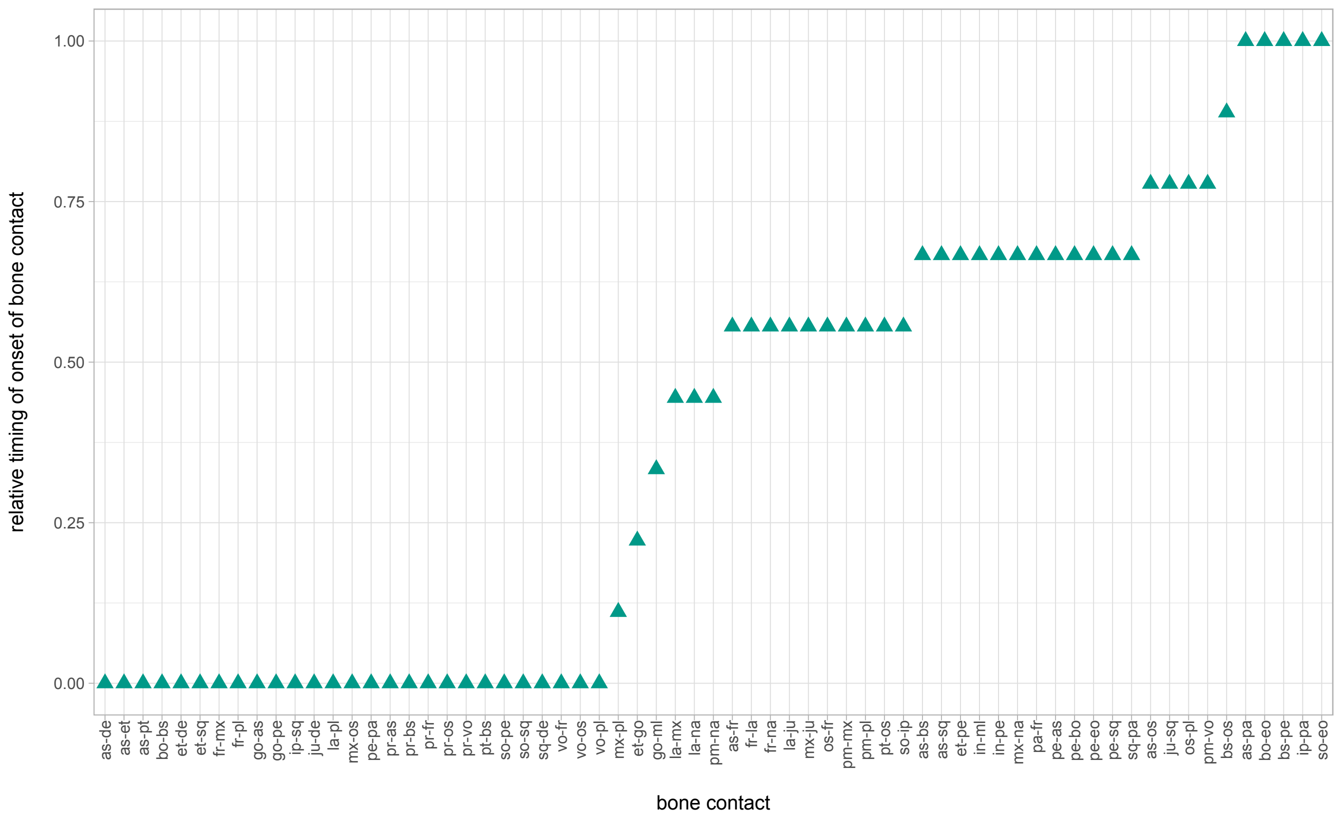

Supplementary Figure 6. Relative timing of onset of bone contacts for the bones of the skull in *S. crassicaudata*.

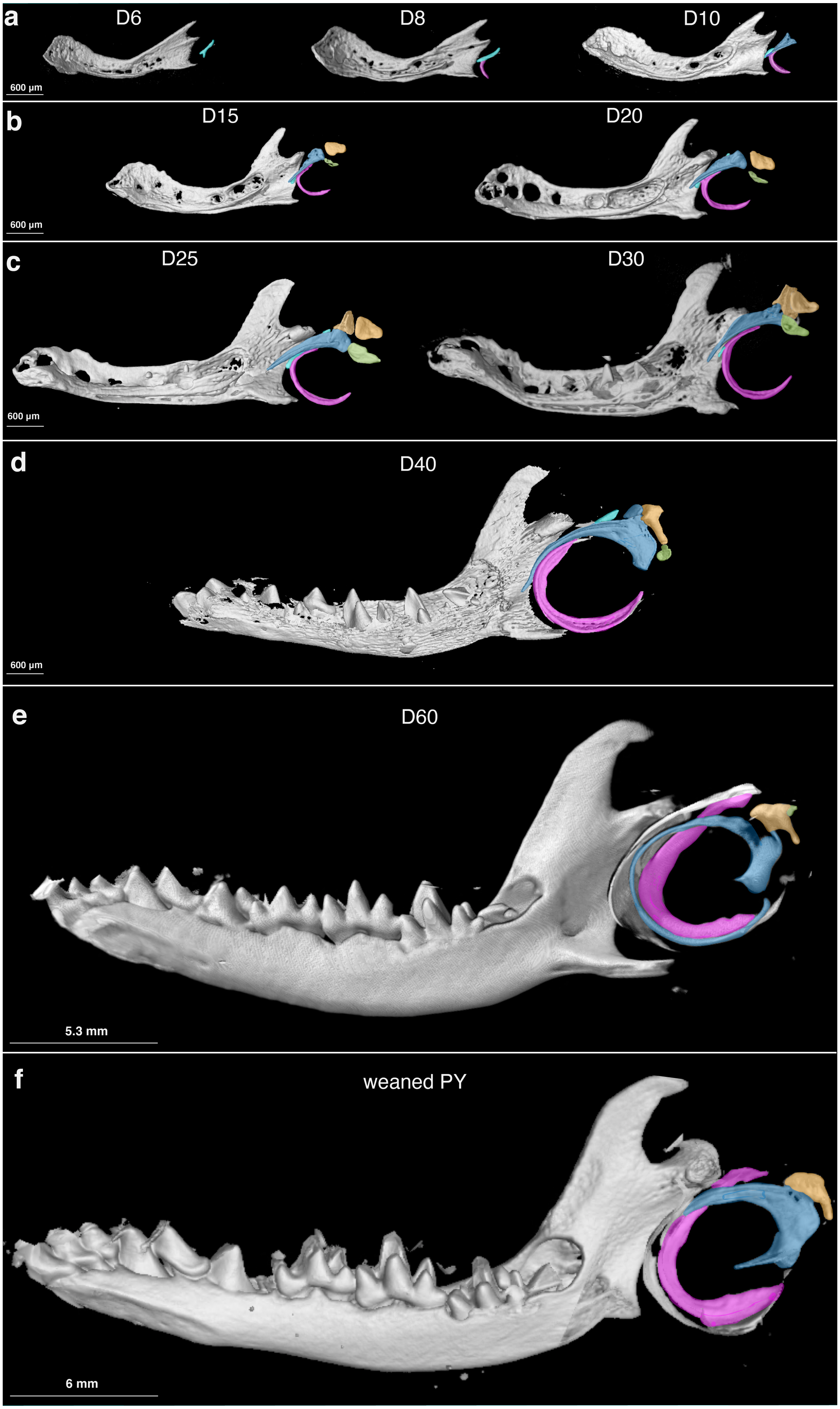

Supplementary Figure 7. Development of the lower jaw and inner ear bones as revealed by microCT in fat-tailed dunnart pouch young. **a** D6, D8 and D10, **b** D15 and D20, **c** D25 and D30, **d** D40, **e** D60, **f** weaned juvenile pouch young. Goniale (light blue), ectotympanic ring (purple), incus (yellow), malleus (blue) and stapes (light green).

Supplementary Tables

Supplementary Table 1. Onset of bone contacts with age and rankings observed in the cranial skeleton of *S. crassicaudata* pouch young.

Bone contact	Age (days)	Rank
ectotympenoid-goniale	5	1
maxilla-palatine	8	2
goniale-malleus	10	3
lacrimal-maxilla	10	3
premaxilla-maxilla	10	3
premaxilla-nasal	15	4
maxilla-nasal	25	5
supraoccipital-interparietal	30	6
parietal-frontal	30	6
frontal-nasal	30	6
lacrimal-jugal	30	6
maxilla-jugal	30	6
frontal-lacrimal	30	6
alisphenoid-frontal	30	6
squamosal-parietal	35	7
alisphenoid-squamosal	35	7
alisphenoid-pterygoid	35	7
lacrimal-palatine	35	7
jugal-squamosal	35	7
petrosal-exoccipital	35	7
petrosal-basioccipital	35	7
petrosal-squamosal	35	7
petrosal-alisphenoid	35	7
incus-malleus	35	7
incus-petrosal	35	7
ectotympic-petrosal	35	7
frontal-maxilla	35	7
frontal-palatine	35	7
interparietal-parietal	60	8
basisphenoid-petrosal	60	8
alisphenoid-parietal	60	8
basioccipital-exoccipital	60	8
supraoccipital-exoccipital	60	8
basioccipital-basisphenoid	w.juv	9
supraoccipital-squamosal	w.juv	9
supraoccipital-petrosal	w.juv	9

Supplementary Table 2. Average weight, head length and crown-rump length for fat-tailed dunnart pouch young.

Age (days)	n	Weight (g)	Head length (mm)	Crown-rump length (mm)
0	9	0.0162 ± 0.0004	2.40 ± 0.05	4.32 ± 0.06
1	3	0.0170 ± 0.0003	2.71 ± 0.03	4.52 ± 0.01
2	2	0.0329 ± 0.0005	2.93 ± 0.005	5.13 ± 0.03
3	4	0.0391 ± 0.0020	3.38 ± 0.18	5.43 ± 0.07
4	8	0.0430 ± 0.0012	3.40 ± 0.13	5.73 ± 0.09
5	4	0.0556 ± 0.0018	4.44 ± 0.08	6.53 ± 0.08
6	2	0.0601 ± 0.0018	4.51 ± 0.18	7.03 ± 0.09
7	2	0.0601 ± 0.0005	4.89 ± 0.07	7.00 ± 0.07
8	2	0.0685 ± 0.0043	4.83 ± 0.24	7.52 ± 0.15
9	2	0.0759 ± 0.0022	5.15	7.5 ± 0.02
10	2	0.0816 ± 0.0063	5.38 ± 0.14	8.79 ± 0.02
15	2	0.1199 ± 0.0131	5.93 ± 0.34	10.02 ± 0.16
20	2	0.1879 ± 0.0048	6.75 ± 0.12	12.51 ± 0.01
25	6	0.2834 ± 0.0041	7.90 ± 0.04	15.76 ± 0.027
30	1	0.3239	8.47	14.09
35	1	0.5531	10.15	20.13
40	1	1.1996	13.84	27.13
50	1	3.432	18.21	38.38
60	1	5.222	21.48	44.62

Supplementary References

1. Iwata, J., Bringas, P. & Chai, Y. Color-coded scanning electron microscopy images of C57BL/6 mouse embryos. *FaceBase Consortium*, https://www.facebase.org/mouseanatomy (Accessed: April 2021).

2. Brinkley, J. F. *et al.* The FaceBase Consortium: a comprehensive resource for craniofacial researchers. *Development* **143**, 2677–2688, 10.1242/dev.135434 (2016).

3. Fawcett, E. The primordial cranium of *Erinaceus europaeus*. *Journal of Anatomy* **52**, 211–250.45 (1918).

4. Nunn, C. L. & Smith, K. K. Statistical analyses of developmental sequences: the craniofacial region in marsupial and placental mammals. *The American Naturalist* **152**, 82–101 (1998).

5. Spiekman, S. N. F. & Werneburg, I. Patterns in the bony skull development of marsupials: high variation in onset of ossification and conserved regions of bone contact. *Sci Rep* **7**, 11, 10.1038/srep43197 (2017).
