# Supplementary material for: Postnatal development in a marsupial model, the fat-tailed dunnart (Sminthopsis crassicaudata; Dasyuromorphia: Dasyuridae)
Source: Commun Biol. 2021 Sep 2;4:1028. doi: 10.1038/s42003-021-02506-2 (PMC8413461; doi:10.1038/s42003-021-02506-2)
Supplement: Supplementary file 2 — Description of Supplementary Files [file 42003_2021_2506_MOESM2_ESM.pdf]

Description of Additional Supplementary Files

File name: Supplementary Movie 1
Video of newborn (D0) fat-tailed dunnart (*S. crassicaudata*) in culture medium.

File name: Supplementary Movie 2
MicroCT volume rendering of a D0 dunnart (*S. crassicaudata*) showing sagittal slices from right to left.

File name: Supplementary Movie 3
MicroCT volume rendering of a D30 dunnart (*S. crassicaudata*) showing sagittal slices from right to left.

File name: Supplementary Movie 4
MicroCT volume rendering of a D35 dunnart (*S. crassicaudata*) showing sagittal slices from right to left.

File name: Supplementary Movie 5
MicroCT volume rendering of a D40 dunnart (*S. crassicaudata*) showing sagittal slices from right to left.

File name: Supplementary Movie 6
MicroCT volume rendering of a D50 dunnart (*S. crassicaudata*) showing sagittal slices from right to left.

File name: Supplementary Movie 7
MicroCT volume rendering of a D60 dunnart (*S. crassicaudata*) showing sagittal slices from right to left.

File name: Supplementary Data 1
Description: Details of specimen collection, preparation and microCT scanning parameters.

File name: Supplementary Data 2
Description: Rankings for the onset of ossification in the skull of *S. crassicaudata* pouch young.

File name: Supplementary Data 3
Description: Rankings for the onset of bone contacts in the skull of S. crassicaudata pouch young.
