# Supplementary material for: Postnatal development in a marsupial model, the fat-tailed dunnart (Sminthopsis crassicaudata; Dasyuromorphia: Dasyuridae)
Source: Commun Biol. 2021 Sep 2;4:1028. doi: 10.1038/s42003-021-02506-2 (PMC8413461; doi:10.1038/s42003-021-02506-2)
Supplement: Supplementary file 13 — Reporting Summary [file 42003_2021_2506_MOESM13_ESM.pdf]

nature research

Corresponding author(s): *Double-blind peer review submissions: write DBPR and your manuscript number here instead of author names.*

Last updated by author(s): *YYYY-MM-DD*

Reporting Summary

Nature Research wishes to improve the reproducibility of the work that we publish. This form provides structure for consistency and transparency in reporting. For further information on Nature Research policies, see our Editorial Policies and the Editorial Policy Checklist.

Statistics

For all statistical analyses, confirm that the following items are present in the figure legend, table legend, main text, or Methods section.

n/a	Confirmed	
[ ]	[x]	The exact sample size (*n*) for each experimental group/condition, given as a discrete number and unit of measurement
[ ]	[x]	A statement on whether measurements were taken from distinct samples or whether the same sample was measured repeatedly
[x]	[ ]	The statistical test(s) used AND whether they are one- or two-sided<br>*Only common tests should be described solely by name; describe more complex techniques in the Methods section.*
[x]	[ ]	A description of all covariates tested
[x]	[ ]	A description of any assumptions or corrections, such as tests of normality and adjustment for multiple comparisons
[ ]	[x]	A full description of the statistical parameters including central tendency (e.g. means) or other basic estimates (e.g. regression coefficient) AND variation (e.g. standard deviation) or associated estimates of uncertainty (e.g. confidence intervals)
[x]	[ ]	For null hypothesis testing, the test statistic (e.g. *F*, *t*, *r*) with confidence intervals, effect sizes, degrees of freedom and *P* value noted<br>*Give P values as exact values whenever suitable.*
[x]	[ ]	For Bayesian analysis, information on the choice of priors and Markov chain Monte Carlo settings
[x]	[ ]	For hierarchical and complex designs, identification of the appropriate level for tests and full reporting of outcomes
[x]	[ ]	Estimates of effect sizes (e.g. Cohen's *d*, Pearson's *r*), indicating how they were calculated

Our web collection on statistics for biologists contains articles on many of the points above.

Software and code

Policy information about availability of computer code

Data collection *Provide a description of all commercial, open source and custom code used to collect the data in this study, specifying the version used OR state that no software was used.*

Data analysis *Provide a description of all commercial, open source and custom code used to analyse the data in this study, specifying the version used OR state that no software was used.*

For manuscripts utilizing custom algorithms or software that are central to the research but not yet described in published literature, software must be made available to editors and reviewers. We strongly encourage code deposition in a community repository (e.g. GitHub). See the Nature Research guidelines for submitting code & software for further information.

Data

Policy information about availability of data

All manuscripts must include a data availability statement. This statement should provide the following information, where applicable:

- Accession codes, unique identifiers, or web links for publicly available datasets
- A list of figures that have associated raw data
- A description of any restrictions on data availability

All X-ray and reconstructed µCT data shown are publicly available through a MorphoSource repository (www.morphosource.org}, Project P1150). Histology slide images are available through a figshare repository (www.figshare.com, Project 111930). For comparison of ossification patterns between species, timing and ranks were obtained from a previous publication dataset by Koyabu et al. 2014.

Field-specific reporting

Please select the one below that is the best fit for your research. If you are not sure, read the appropriate sections before making your selection.

- [x] Life sciences
- [ ] Behavioural & social sciences
- [ ] Ecological, evolutionary & environmental sciences

For a reference copy of the document with all sections, see nature.com/documents/nr-reporting-summary-flat.pdf

Life sciences study design

All studies must disclose on these points even when the disclosure is negative.

Sample size	This is a descriptive study with no statistical analyses performed. As pouch young specimens were precisely staged and given the use of a native marsupial model we kept the sample size small with 1 or 2 pouch young microCT scanned per age. For generating mean data for weights, head lengths and crown-rump lengths sample size was based on litter size and age and generation of pouch for other projects in the laboratory. Again, this was to keep animal numbers low in accordance with our ethical requirements.
Data exclusions	No data were excluded.
Replication	Two D0 specimens were microCT scanned to confirm lack of bone. Replicated data for average weight, head length and crown-rump length was based on litter size and age.
Randomization	This is not relevant to our study. This study was a descriptive study describing presence/absence of bones in the cranial and postcranial skeleton.
Blinding	Blinding was not relevant to our study because we were not performing any treatments.

Reporting for specific materials, systems and methods

We require information from authors about some types of materials, experimental systems and methods used in many studies. Here, indicate whether each material, system or method listed is relevant to your study. If you are not sure if a list item applies to your research, read the appropriate section before selecting a response.

Materials & experimental systems

n/a	Involved in the study	
x		Antibodies
x		Eukaryotic cell lines
x		Palaeontology and archaeology
	x	Animals and other organisms
x		Human research participants
x		Clinical data
x		Dual use research of concern

Methods

n/a	Involved in the study	
x		ChIP-seq
x		Flow cytometry
x		MRI-based neuroimaging

Animals and other organisms

Policy information about studies involving animals; ARRIVE guidelines recommended for reporting animal research

Laboratory animals	Sminthopsis crassicaudata (the fat-tailed dunnart), age P0-P70, male and female specimens.
Wild animals	Study did not include wild animals.
Field-collected samples	Study did not involve samples collected in the field.
Ethics oversight	All animal procedures, including laboratory breeding, were conducted in accordance with the current Australian Code for the Care and Use of Animals for Scientific Purposes and were approved by The University of Melbourne Animal Ethics Committee (AEC: 1513686.2) and with the appropriate Wildlife Permit (number 10008652) from the Department of Environment, Land, Water and Planning.

Note that full information on the approval of the study protocol must also be provided in the manuscript.
